# Quercetin Sensitizes Retinoblastoma Cells to Mitomycin C Through Transcriptional Modulation of p53-Regulated Apoptotic Genes: A Preclinical Study

**DOI:** 10.3390/ph19040545

**Published:** 2026-03-28

**Authors:** Erkan Duman, Aydın Maçin, İlhan Özdemir, Şamil Öztürk, Mehmet Cudi Tuncer

**Affiliations:** 1 Department of Ophthalmology, WestEye Private Hospital, Erbil 44001, Iraq; ophthalmo48@outlook.com; 2 Department of Ophthalmology, Diyarbakır Private Batı Hospital, 21100 Diyarbakır, Turkey; draydinmacin@outlook.com; 3 Department of Histology and Embryology, Faculty of Medicine, Kahramanmaraş Sütçü İmam University, 46040 Kahramanmaraş, Turkey; ilhanozdemir25@yandex.com; 4 Vocational School of Health Services, Çanakkale Onsekiz Mart University, 17100 Çanakkale, Turkey; ozturksamil@outlook.com; 5 Department of Anatomy, Faculty of Medicine, Dicle University, 21280 Diyarbakır, Turkey

**Keywords:** quercetin, mitomycin C, retinoblastoma cells, apoptosis induction, combination index

## Abstract

**Background/Objectives**: Retinoblastoma represents the most common intraocular malignancy in childhood; however, the clinical applicability of mitomycin C (MMC) is restricted by dose-dependent ocular toxicity. Consequently, the development of pharmacological strategies that sensitize tumor cells to MMC while allowing dose reduction remains an unmet therapeutic objective. In this context, quercetin, a bioactive flavonoid with pleiotropic anticancer properties, has emerged as a potential chemosensitizing agent. **Methods**: Human retinoblastoma cell lines Y79 and WERI-Rb1 were exposed to MMC and quercetin, administered either individually or in fixed-ratio combinations. Cytotoxic responses were quantified through dose–response modeling and IC_50_ determination following 24 and 48 h of treatment. Drug–drug interactions were quantitatively characterized using the Chou–Talalay combination index (CI) approach and isobologram analysis. Cell cycle distribution was assessed by propidium iodide (PI)-based flow cytometric analysis to evaluate treatment-associated alterations in cell cycle progression. Apoptotic cell death was assessed by Annexin V-FITC/PI flow cytometry, while transcriptional modulation of genes associated with apoptosis, cell cycle regulation, and oxidative stress (BAX, BCL-2, TP53, CASP3, CDKN1A, and HMOX1) was evaluated by qRT-PCR. Modulation of tumor-supportive signaling was examined by measuring VEGF and IL-6 secretion. Translational relevance was further investigated using a three-dimensional (3D) tumor spheroid model, and the functional contribution of reactive oxygen species (ROS) was interrogated through N-acetyl-L-cysteine (NAC) rescue experiments. **Results**: Quercetin significantly enhanced the cytotoxic activity of MMC in both retinoblastoma cell lines, with CI values below 1 across IC_50_–IC_90_ effect levels, indicating a synergistic pharmacological interaction. PI–FACS analysis revealed that combined MMC and quercetin treatment induced a pronounced accumulation of cells in the G2/M phase, consistent with cell cycle arrest, with a more marked effect observed in Y79 cells compared with WERI-Rb1 cells. Combination treatment resulted in a pronounced increase in apoptotic cell populations compared with single-agent exposure and triggered a coordinated pro-apoptotic transcriptional response, characterized by increased expression of BAX, TP53, CASP3, CDKN1A, and HMOX1, alongside suppression of BCL-2 and a marked shift in the BAX/BCL-2 ratio. Concurrently, VEGF and IL-6 secretion were significantly reduced, reflecting attenuation of pro-angiogenic and pro-inflammatory signaling. Notably, synergistic cytotoxicity was maintained in 3D tumor spheroids, where combined treatment induced spheroid shrinkage, architectural disruption, and reduced viability. NAC pretreatment diminished ROS accumulation and partially restored cell viability, indicating that oxidative stress contributes to, but does not solely account for, the observed synergistic cytotoxic effect. **Conclusions**: Collectively, these findings indicate that quercetin appears to function as an effective chemosensitizing adjuvant to MMC in retinoblastoma models, through transcriptional changes consistent with p53-associated apoptotic signaling at the transcriptional level, G2/M cell cycle arrest, and partial involvement of ROS-related cellular stress responses, along with suppression of tumor-supportive signaling pathways. The preservation of synergistic activity in 3D tumor spheroids supports the potential preclinical relevance of this combination. However, these findings are based on transcriptional and phenotypic analyses and should be interpreted as hypothesis-generating, requiring further validation through protein-level and in vivo studies before translational application.

## 1. Introduction

Ocular malignancies constitute a clinically important group of cancers due to their capacity to cause permanent visual impairment and, in advanced stages, life-threatening systemic spread [1]. Within this spectrum, uveal melanoma represents the most prevalent primary intraocular malignancy in adults, whereas retinoblastoma is the most commonly diagnosed intraocular cancer during childhood [1,2]. Although substantial progress has been achieved in local treatment strategies, including surgical intervention, radiotherapy, and chemotherapy, clinical outcomes remain suboptimal in advanced disease, therapy-resistant tumors, and cases associated with an elevated risk of metastasis [3,4]. Notably, the absence of effective systemic treatment options following hepatic metastasis in uveal melanoma highlights a critical unmet need for the development of more effective therapeutic approaches [5].

MMC is a DNA-alkylating antineoplastic agent that exerts its cytotoxic effects primarily through the induction of DNA cross-linking, leading to cell cycle arrest and apoptotic cell death. In the field of ocular oncology, MMC is widely employed as an adjuvant therapeutic agent, particularly in the treatment of ocular surface squamous neoplasia and within localized chemotherapy regimens for selected intraocular tumors [6]. Nevertheless, the broader clinical utility of MMC is limited by several factors, including dose-dependent local toxicity, the risk of systemic exposure, and the emergence of drug resistance, especially under conditions of prolonged administration [7]. As a result, considerable research efforts have focused on identifying safe and effective combination strategies capable of enhancing MMC antitumor efficacy while permitting dose reduction.

In recent years, naturally derived flavonoids have attracted considerable attention as bioactive compounds with broad applications in cancer prevention and therapy. These polyphenolic molecules, widely present in plant-based foods, are characterized by diverse biological activities, including antioxidant, anti-inflammatory, anti-mutagenic, and anti-carcinogenic effects, as well as the ability to modulate key cellular enzymes and signaling processes [8]. Accumulating evidence indicates that flavonoids exert chemopreventive and cytotoxic effects through multiple mechanisms rather than a single molecular target, influencing pathways involved in cell proliferation, apoptosis, and tumor progression [9].

Mechanistically, flavonoids and related phenolic compounds have been shown to induce apoptosis, promote cell cycle arrest, regulate carcinogen metabolism, and inhibit critical processes such as cell adhesion, migration, and proliferation, largely through modulation of intracellular signaling networks [10]. In particular, these compounds are capable of targeting major oncogenic pathways, including PI3K/AKT, MAPK, NF-κB, and Wnt/β-catenin signaling, while simultaneously regulating apoptosis-related proteins and cell cycle regulators. Such multi-target activity is increasingly recognized as a key advantage in overcoming the complexity of cancer biology.

Importantly, emerging evidence suggests that flavonoids can act as effective adjuvant agents in combination-based therapeutic strategies. Studies on structurally related compounds, such as genistein, have demonstrated their ability to enhance the efficacy of conventional chemotherapeutic agents through synergistic interactions, while modulating apoptosis, angiogenesis, and cell cycle progression [11]. These findings highlight the potential of flavonoids not only as standalone anticancer agents but also as chemosensitizers capable of improving therapeutic outcomes and reducing treatment-associated limitations.

In parallel, increasing attention has been directed toward naturally derived bioactive compounds as adjuncts to conventional chemotherapeutic regimens, driven by their potential to potentiate anticancer activity while limiting treatment-related adverse effects [12,13]. Quercetin (3,3′,4′,5,7-pentahydroxyflavone), a dietary flavonoid widely distributed in fruits and vegetables, has gained prominence as a candidate molecule in this context. In addition to its well-established antioxidant and anti-inflammatory properties, quercetin has been shown to exhibit antiproliferative, pro-apoptotic, anti-angiogenic, and anti-metastatic effects across a variety of cancer models [14,15]. These effects are mediated through the regulation of cell cycle-associated proteins, suppression of key pro-survival signaling pathways such as PI3K/AKT, MAPK, and NF-κB, and activation of intrinsic apoptotic cascades [16]. Importantly, quercetin’s favorable toxicity profile enhances its suitability for use in combination-based therapeutic strategies. Consistent with this rationale, synergistic interactions between quercetin and established chemotherapeutic agents, including cisplatin, doxorubicin, and 5-fluorouracil, have been reported in multiple malignancies [17].

Despite accumulating evidence supporting the anticancer potential of quercetin, the therapeutic relevance and mechanistic basis of its combination with MMC remain insufficiently defined, particularly in ocular malignancies where treatment options are limited and dose-limiting toxicity remains a major concern. It is hypothesized that quercetin may potentiate MMC-induced cytotoxicity by enhancing DNA damage-associated signaling, inducing G2/M cell cycle checkpoint arrest, lowering apoptotic thresholds, and partially engaging ROS-associated stress responses. Accordingly, the present study was designed to quantitatively assess the cytotoxic and antiproliferative effects of MMC in combination with quercetin in the retinoblastoma cell lines Y79 and WERI-Rb1. Drug–drug interactions were systematically evaluated using established mathematical models of synergy, while the underlying molecular mechanisms were explored with particular emphasis on cell cycle regulation, apoptosis, and oxidative stress-associated pathways. By integrating analyses performed in both two-dimensional monolayer cultures and three-dimensional tumor spheroid models, this study aims to provide mechanistic insight into the potential role of quercetin as a chemosensitizing adjuvant for enhancing MMC efficacy in ocular cancer therapy.

## 2. Results

### 2.1. Differential Cytotoxic Response of Retinoblastoma Cells to MMC

To delineate cell line-dependent differences in sensitivity to MMC, Y79 and WERI-Rb1 retinoblastoma cells were treated with increasing concentrations of MMC (0.1–10 µM) for 48 h, and cell viability was quantified using the CCK-8 assay. Exposure to MMC resulted in a pronounced, concentration-dependent decline in cell viability in both retinoblastoma cell lines (Figure S1).

Across the tested concentration range after 48 h of exposure, Y79 cells consistently exhibited a greater loss of viability compared with WERI-Rb1 cells. At an MMC concentration of 1 µM, the viability of Y79 cells was reduced to approximately 45–50% of control levels, whereas WERI-Rb1 cells retained nearly 70–75% viability. This differential response became increasingly evident at higher drug concentrations. Specifically, Y79 cell viability declined to below 30% at 5 µM and further decreased to approximately 15–20% at 10 µM MMC. In contrast, WERI-Rb1 cells demonstrated substantially higher survival rates at the corresponding concentrations.

Analysis of dose–response curves further revealed that Y79 cells exhibited a lower half-maximal inhibitory concentration (IC_50_) relative to WERI-Rb1 cells, reflecting a greater intrinsic susceptibility to MMC-induced cytotoxicity. Conversely, the response profile of WERI-Rb1 cells was comparatively attenuated, consistent with a more resistant cellular phenotype.

Overall, these results indicate distinct cytotoxic response patterns to MMC between Y79 and WERI-Rb1 retinoblastoma cells. Accordingly, these two cell lines serve as complementary experimental models representing MMC-sensitive and relatively less-responsive retinoblastoma phenotypes, respectively (Figure S1).

### 2.2. Effects of MMC and Quercetin on Cell Viability Following Short-Term Exposure

**Figure 1:**
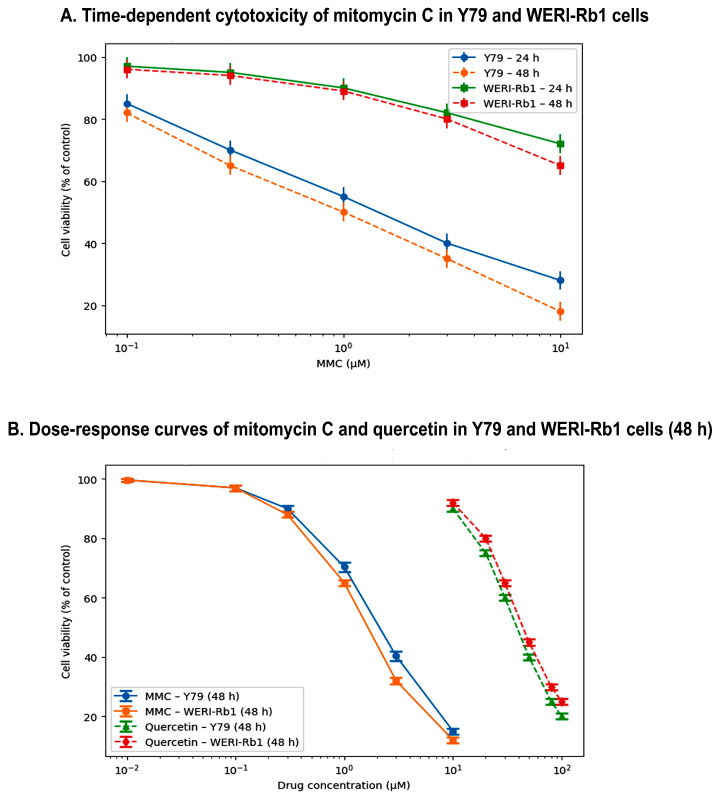
Cytotoxic effects of MMC and quercetin in retinoblastoma cell lines. (**A**) Time-dependent cytotoxicity of Mitomycin C in Y79 and WERI-Rb1 cells following 24 h and 48 h treatment. Increasing MMC concentrations resulted in a progressive reduction in cell viability, with a stronger effect observed after 48 h exposure in both cell lines. (**B**) Dose–response curves of Mitomycin C and quercetin in Y79 and WERI-Rb1 cells after 48 h treatment. Cell viability was determined using the CCK-8 assay and expressed as a percentage of control cells. Data are presented as mean ± standard deviation (SD) from three independent experiments (*n* = 3). Statistical analysis was performed using one-way analysis of variance (ANOVA) followed by Tukey’s multiple comparison test, and differences were considered statistically significant at *p* < 0.05.

To examine the early cytotoxic responses to MMC and quercetin, cell viability was evaluated after 24 h of treatment in Y79 and WERI-Rb1 retinoblastoma cell lines. Short-term exposure to either compound resulted in detectable, concentration-dependent reductions in cell viability, with statistically significant effects predominantly observed at the highest concentrations tested (Figure 1A).

In Y79 cells, exposure to 10 µM MMC reduced cell viability to 65.4 ± 3.2% relative to untreated controls, whereas treatment with 100 µM quercetin decreased viability to 58.9 ± 4.1%. A comparable pattern was observed in WERI-Rb1 cells, in which viability values following treatment with 10 µM MMC and 100 µM quercetin were 62.1 ± 3.8% and 61.5 ± 4.7%, respectively. These findings indicate that both agents exert moderate cytotoxic effects under short-term exposure conditions, with quercetin producing a slightly more pronounced early response in Y79 cells.

By contrast, treatment with lower concentrations of MMC (0.1–1 µM) and quercetin (5–25 µM) resulted in minimal alterations in cell viability in both cell lines following 24 h of exposure. At these dose ranges, viability remained close to control levels, suggesting that short-term treatment is insufficient to elicit substantial cytotoxicity at submaximal concentrations.

Collectively, these observations indicate that the cytotoxic activity of MMC is strongly time-dependent and requires extended exposure to achieve maximal effects. The limited impact observed at lower concentrations during the 24 h treatment period underscores the critical role of exposure duration in shaping cellular responses to MMC and quercetin (Figure 1A).

Extending the treatment duration to 48 h resulted in a marked increase in the cytotoxic efficacy of both MMC and quercetin in Y79 and WERI-Rb1 cells. Dose–response analysis revealed a clear time- and concentration-dependent reduction in cell viability for both agents (Figure 1B).

In Y79 cells, the calculated IC_50_ values following 48 h of treatment were 2.1 µM for MMC and 28.5 µM for quercetin. Similarly, WERI-Rb1 cells exhibited comparable sensitivity to MMC, with an IC_50_ value of 1.8 µM, while demonstrating a relatively more resistant profile to quercetin (IC_50_: 35.2 µM). Analysis of the dose–response curves indicated that MMC exerted a pronounced inhibitory effect on cell viability beginning at concentrations of approximately 1 µM, whereas quercetin-induced cytotoxicity became evident at concentrations of approximately 25 µM and above.

At higher concentrations, particularly 5 µM MMC and ≥80 µM quercetin, cell viability in both Y79 and WERI-Rb1 cells decreased to below 30%, indicating pronounced cytotoxic activity. These findings suggest that although MMC is effective at relatively low micromolar concentrations, such doses may be associated with an elevated risk of local toxicity in clinical settings. By contrast, quercetin administered as a single agent required comparatively higher concentrations to induce a similar extent of cytotoxicity.

Collectively, these time- and dose-dependent cytotoxicity profiles support the rationale for combination-based therapeutic strategies, suggesting that the concomitant use of MMC and quercetin at lower, potentially safer concentrations may preserve or enhance antitumor efficacy while reducing dose-related toxicity (Figure 1B).

### 2.3. Quantitative Assessment of Synergistic Interaction Between MMC and Quercetin

To quantitatively characterize the interaction between MMC and quercetin, combination effects were evaluated using the Chou–Talalay CI method together with isobologram analysis. Fixed-ratio combination treatment was applied at an approximate MMC:quercetin ratio of 1:15, corresponding to IC_50_-derived concentrations of 2.1 µM MMC with 28.5 µM quercetin in Y79 cells and 1.8 µM MMC with 35.2 µM quercetin in WERI-Rb1 cells, as determined from single-agent IC_50_ values, and cytotoxic effects were assessed following 48 h of exposure.

**Figure 2:**
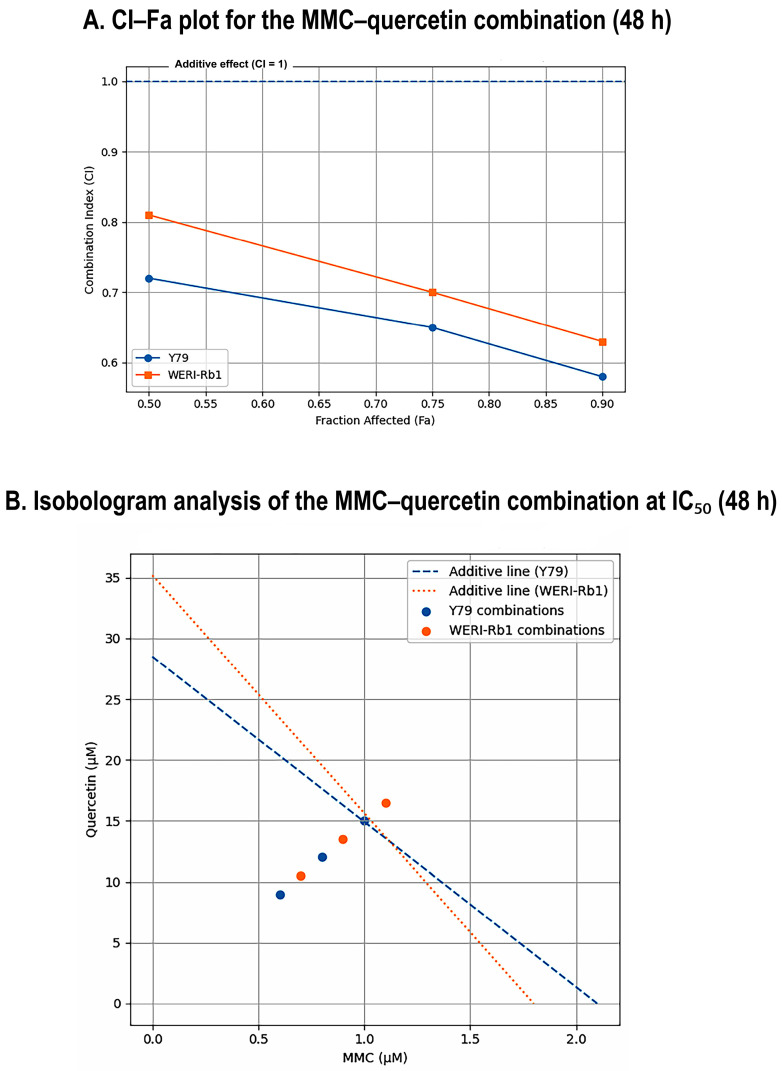
Evaluation of the interaction between MMC and quercetin in retinoblastoma cells using combination index and isobologram analyses after 48 h treatment. (**A**) CI–Fa plot generated using the Chou–Talalay method for Y79 and WERI-Rb1 retinoblastoma cells treated with MMC and quercetin at a fixed MMC:quercetin ratio of approximately 1:15. CI values were calculated at different effect levels (Fa = 0.50, 0.75, and 0.90). The horizontal dashed blue line represents the additive effect threshold (CI = 1), serving as a reference to distinguish between synergistic (CI < 1) and antagonistic (CI > 1) interactions. CI values below 1 indicate synergistic interactions between the two agents. The MMC–quercetin combination demonstrated synergistic effects across the evaluated effect levels in both cell lines, with a more pronounced synergistic profile observed in Y79 cells. (**B**) Isobologram analysis of the MMC–quercetin combination at the IC_50_ effect level in Y79 and WERI-Rb1 cells after 48 h of treatment. The line of additivity represents the theoretical dose combinations expected for an additive interaction. Experimental combination points located below the additivity line indicate synergistic interactions between MMC and quercetin. In both retinoblastoma cell lines, the observed combination points fell within the synergy region, confirming a synergistic interaction.

In Y79 cells, combination treatment yielded CI values of 0.72, 0.65, and 0.58 at the IC_50_, IC_75_, and IC_90_ effect levels, respectively, indicating a strong synergistic interaction that increased progressively with higher degrees of growth inhibition. Similarly, WERI-Rb1 cells demonstrated synergistic responses to the combination treatment, with CI values of 0.81, 0.70, and 0.63 at the corresponding effect levels. These results indicate that the MMC–quercetin combination consistently produced CI values below 1 across multiple fractional effect levels in both retinoblastoma cell lines (Figure 2A).

Isobologram analysis further corroborated these findings. In both Y79 and WERI-Rb1 cells, the combination data points were positioned below the line of additivity, confirming synergistic interactions between MMC and quercetin under the tested conditions. The deviation toward the synergy region was more pronounced in Y79 cells, suggesting a relatively stronger synergistic interaction compared with WERI-Rb1 cells, although clear synergism was observed in both retinoblastoma models (Figure 2B).

### 2.4. PI–FACS Analysis Reveals G2/M Cell Cycle Arrest Induced by MMC and Quercetin Combination

**Figure 3:**
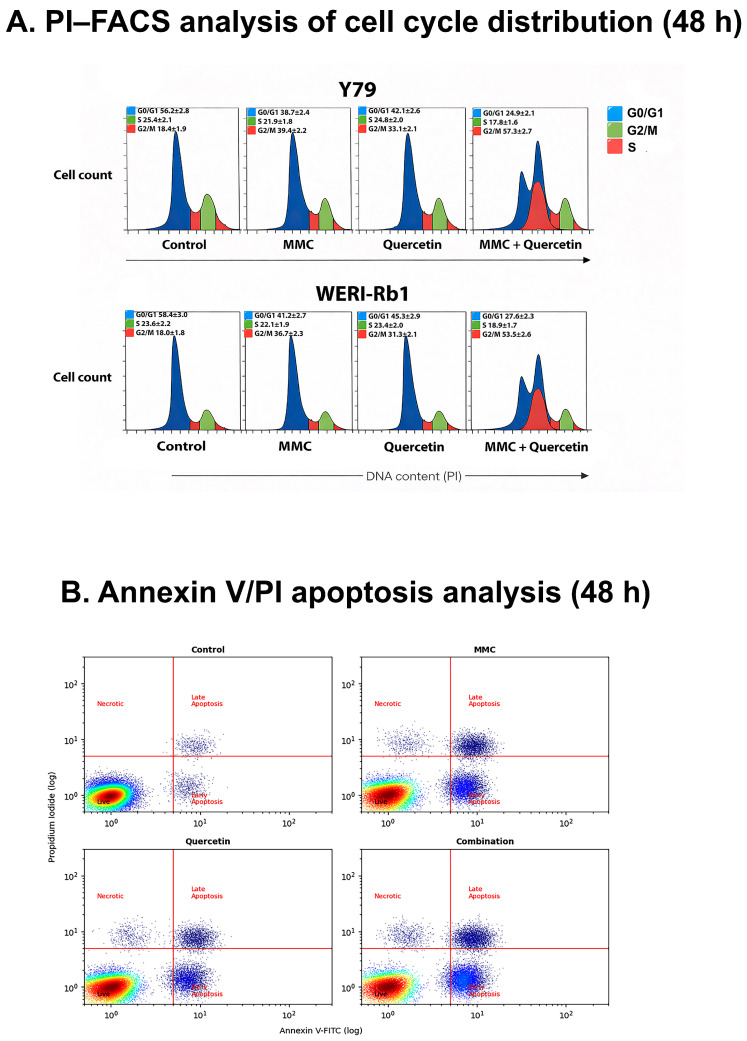
Cell cycle arrest and apoptosis induction in retinoblastoma cells following MMC and quercetin treatment. (**A**) PI–FACS analysis of cell cycle distribution in retinoblastoma cells after 48 h treatment. PI-based flow cytometric analysis was performed to evaluate cell cycle distribution in Y79 and WERI-Rb1 retinoblastoma cell lines following treatment with MMC, quercetin, or their combination. Representative DNA content histograms display the relative proportions of cells in the G0/G1 (blue), S (red), and G2/M (green) phases. Percentages shown indicate the mean ± SD of cells in each cell cycle phase derived from three independent experiments. Compared with control and single-agent treatments, combined MMC and quercetin exposure resulted in a pronounced accumulation of cells in the G2/M phase in both cell lines, with a more marked effect observed in Y79 cells. Histograms were analyzed using FlowJo software (version 10; BD Biosciences, Ashland, OR, USA). (**B**) Annexin V-FITC/PI apoptosis analysis in Y79 retinoblastoma cells after 48 h treatment. Representative Annexin V-FITC/PI flow cytometry density plots illustrate apoptotic cell death in Y79 cells following treatment with MMC, quercetin, or their combination. Cells were classified into live (Annexin V^−^/PI^−^), early apoptotic (Annexin V^+^/PI^−^), late apoptotic (Annexin V^+^/PI^+^), and necrotic (Annexin V^−^/PI^+^) populations. Treatment with MMC or quercetin alone increased apoptotic cell fractions compared with control cells, whereas combination treatment resulted in a markedly enhanced apoptotic response. Each plot represents 20,000 acquired events from one of three independent experiments.

To investigate whether the antiproliferative effects of MMC and quercetin were associated with alterations in cell cycle progression, PI-based flow cytometric analysis was performed after 48 h of treatment (Figure 3A).

In Y79 cells, control samples displayed a typical cell cycle profile characterized by a predominant G0/G1 population with smaller S and G2/M fractions. Treatment with MMC or quercetin alone resulted in moderate redistribution of the cell cycle, with an increase in the G2/M population compared to control. Notably, combined MMC and quercetin treatment caused a pronounced accumulation of cells in the G2/M phase, accompanied by a visible reduction in the G0/G1 fraction, indicating effective G2/M checkpoint arrest (Figure 3A, upper panel).

Similarly, WERI-Rb1 cells exhibited a comparable but less pronounced response. While single-agent treatments induced modest changes in cell cycle distribution, combination treatment led to a clear enrichment of the G2/M population relative to control, consistent with partial cell cycle arrest. The magnitude of this effect was lower than that observed in Y79 cells, reflecting the relatively higher resistance of WERI-Rb1 cells (Figure 3A, lower panel).

Overall, PI–FACS analysis demonstrated that combined MMC and quercetin treatment induces pronounced G2/M phase arrest in retinoblastoma cells, suggesting an association between cell cycle dysregulation and the observed antiproliferative and pro-apoptotic effects (Figure 3A). Quantitative analysis revealed a marked accumulation of cells in the G2/M phase in Y79 cells, with the G2/M fraction increasing from 18.4 ± 1.9% in control cells to 57.3 ± 2.7% following combination treatment, accompanied by a corresponding reduction in the G0/G1 population. In WERI-Rb1 cells, although the magnitude of G2/M accumulation was comparatively lower, combined treatment still significantly increased the G2/M fraction from 18.0 ± 1.8% to 53.5 ± 2.6%, indicating a partial yet robust G2/M arrest.

### 2.5. Induction of Apoptotic Cell Death by MMC and Quercetin Combination Treatment

Apoptotic cell death was assessed by flow cytometric analysis using Annexin V-FITC/PI double staining following 48 h of treatment. Combined exposure to MMC and quercetin resulted in a marked increase in apoptotic cell populations in both retinoblastoma cell lines compared with control conditions and single-agent treatments (Figure 3B).

In Y79 cells, the proportion of apoptotic cells in the control group was 5.2%. Treatment with MMC or quercetin alone significantly increased the apoptotic fraction to 36.1% and 28.7%, respectively (*p* < 0.01 vs. control). Notably, combination treatment further elevated the apoptotic cell ratio to 43%, representing a significantly greater apoptotic response compared with both the control group (*p* < 0.001) and each single-agent treatment (*p* < 0.01).

These results indicate that although MMC and quercetin individually induce apoptosis in Y79 cells, their combined administration produces a substantially enhanced apoptotic effect, consistent with the synergistic cytotoxic interaction observed in cell viability and CI analyses (Figure 3B).

**Figure 4:**
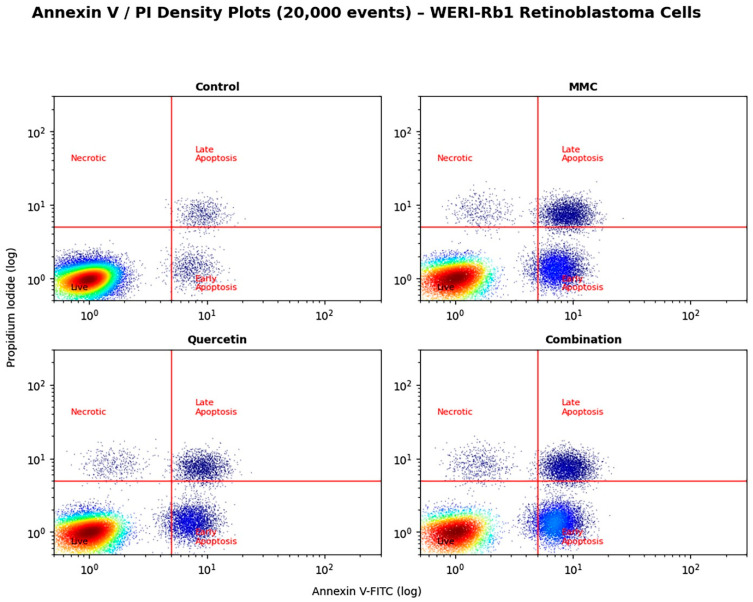
Representative Annexin V-FITC/PI Flow Cytometry Density Plots Illustrating Apoptotic Cell Death in WE-RI-Rb1 Retinoblastoma Cells Following 48 h Treatment. Representative Annexin V-FITC/PI flow cytometry density plots depict apoptotic cell death in WE-RI-Rb1 retinoblastoma cells following 48 h of treatment with MMC, quercetin, or their combination. Based on fluorescence characteristics, cells were categorized as viable (Annexin V^−^/PI^−^, lower left quadrant), early apoptotic (Annexin V^+^/PI^−^, lower right quadrant), late apoptotic (Annexin V^+^/PI^+^, upper right quadrant), or necrotic (Annexin V^−^/PI^+^, upper left quadrant). Compared with control conditions and single-agent treatments, combination treatment produced a pronounced increase in apoptotic cell fractions. Each panel corresponds to 20,000 acquired events obtained from one of three independent experiments.

In WERI-Rb1 cells, apoptotic cells constituted 7.1% of the population under control conditions. Administration of MMC or quercetin as individual treatments resulted in a significant expansion of the apoptotic fraction, corresponding to 42.4% and 30.9%, respectively (*p* < 0.01 vs. control). Notably, co-treatment with MMC and quercetin produced a further escalation of apoptotic cell death, with the apoptotic ratio increasing to 52.7%. This response showed statistically significant differences when compared with both untreated control cells (*p* < 0.001) and the respective single-agent treatment groups (*p* < 0.05) (Figure 4).

### 2.6. Modulation of Signaling Molecules in the Cellular Microenvironment

In addition to evaluating apoptotic and cell cycle-related mechanisms, the secretion of VEGF and IL-6 was examined to explore potential effects of MMC and quercetin on tumor-associated inflammatory and angiogenic signaling pathways. Both cytokines are known to contribute to tumor progression in retinoblastoma by promoting angiogenesis, inflammatory responses, and tumor microenvironment remodeling. Therefore, assessing VEGF and IL-6 levels provided complementary information regarding whether the combined treatment could modulate tumor-associated signaling beyond direct cytotoxic effects.

**Figure 5:**
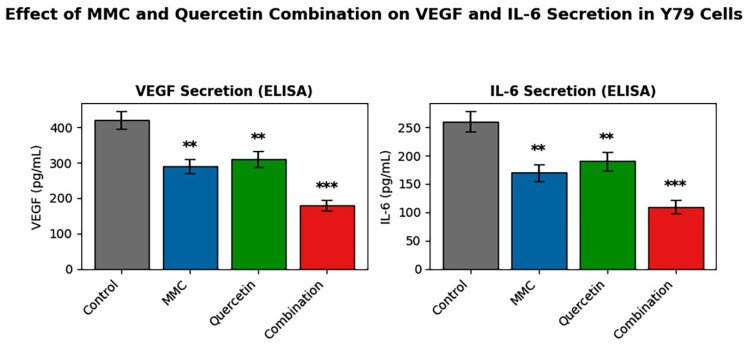
Effects of MMC and Quercetin on VEGF and Interleukin-6 (IL-6) Secretion in Y79 Cells. Levels of VEGF and interleukin-6 (IL-6) in cell culture supernatants were determined by ELISA following 48 h of treatment with MMC, quercetin, or their combination in Y79 cells. Treatment with either MMC or quercetin alone significantly decreased VEGF secretion relative to control cells, whereas combined treatment produced a more pronounced reduction in both VEGF and IL-6 levels. Data are presented as mean ± SD from three independent experiments. ** *p* < 0.01 vs. control; *** *p* < 0.001 vs. control and corresponding single-agent treatments.

Given the more robust and reproducible secretory profile of Y79 cells under the applied experimental conditions, cytokine analyses were confined to this cell line. Enzyme-linked immunosorbent assay (ELISA) measurements revealed marked alterations in pro-angiogenic and pro-inflammatory mediator profiles in Y79 cells following exposure to MMC and quercetin (Figure 5).

Under control conditions, basal secretion of vascular endothelial growth factor (VEGF) was quantified at 412 ± 35 pg/mL. Administration of MMC or quercetin as single agents led to significant decreases in VEGF release, reducing concentrations to 298 ± 27 pg/mL and 325 ± 30 pg/mL, respectively (*p* < 0.01 vs. control). In contrast, combined treatment with MMC and quercetin produced a substantially greater suppression of VEGF secretion, with levels declining to 186 ± 22 pg/mL. This decrease was statistically significant when compared with both untreated control cultures and the corresponding single-agent treatment groups (*p* < 0.001).

A comparable pattern was observed for interleukin-6 (IL-6). Baseline IL-6 concentrations in control culture supernatants were measured at 168 ± 18 pg/mL, whereas combination treatment reduced IL-6 secretion to 92 ± 14 pg/mL, representing a statistically significant reduction relative to control conditions (*p* < 0.001).

### 2.7. Regulation of Apoptosis- and Stress-Related Gene Expression

**Figure 6:**
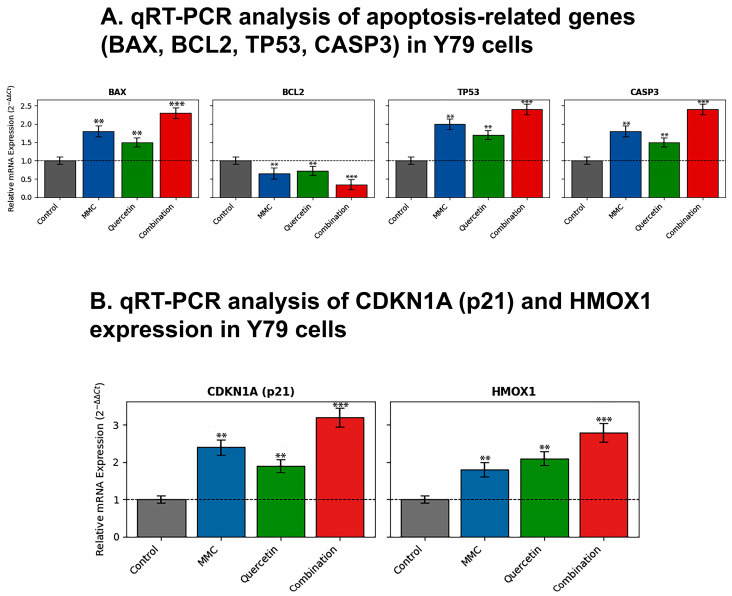
qRT-PCR analysis of apoptosis-, cell cycle-, and stress-related gene expression in Y79 retinoblastoma cells following MMC and quercetin treatment: (**A**) Relative mRNA expression levels of the apoptosis-related genes BAX, BCL2, TP53, and CASP3 in Y79 cells were examined by quantitative real-time polymerase chain reaction (qRT-PCR), normalized to GAPDH, and expressed as fold changes relative to the control group. The dashed horizontal line represents the baseline control level (set to 1), serving as a reference for relative gene expression changes. Compared with control conditions and single-agent treatments, combination treatment with MMC and quercetin resulted in a pronounced increase in the expression of the pro-apoptotic genes BAX, TP53, and CASP3, while concurrently inducing a significant reduction in the expression of the anti-apoptotic gene BCL2. (**B**) Relative mRNA expression levels of CDKN1A (p21) and HMOX1 in Y79 cells were assessed by quantitative real-time polymerase chain reaction (qRT-PCR), normalized to GAPDH, and expressed as fold changes relative to the control group. The dashed horizontal line represents the baseline control level (set to 1), serving as a reference for relative gene expression changes. Compared with control conditions and single-agent treatments, combined exposure to MMC and quercetin produced a pronounced increase in the expression of both CDKN1A and HMOX1. Data are presented as mean ± SD from three independent experiments. ** *p* < 0.01 vs. control; *** *p* < 0.001 vs. control and corresponding single-agent treatments.

Quantitative real-time polymerase chain reaction (qRT-PCR) was employed to assess transcriptional changes in apoptosis- and stress-associated genes following exposure to MMC, quercetin, or their combination. Gene expression values were normalized to GAPDH and reported as fold changes relative to the control condition (Figure 6A).

In Y79 cells, combined treatment with MMC and quercetin led to a pronounced induction of the pro-apoptotic gene BAX, with expression levels reaching a 2.9-fold increase compared with control cells. By comparison, treatment with MMC or quercetin alone produced more limited elevations in BAX expression, corresponding to 1.8-fold and 1.6-fold increases, respectively (*p* < 0.01 vs. control). A similar expression pattern was observed for the tumor suppressor gene TP53. Specifically, combination treatment resulted in a significant increase in TP53 expression, attaining a 3.2-fold elevation, whereas MMC and quercetin administered individually yielded smaller increases of 1.9-fold and 1.7-fold, respectively (*p* < 0.01 vs. control).

In Y79 cells, combination treatment was associated with a significant induction of CDKN1A (p21), a cyclin-dependent kinase inhibitor involved in cell cycle regulation, resulting in a 3.1-fold increase relative to control cells (*p* < 0.001).

Concurrently, expression of the stress-responsive gene HMOX1, which plays a role in cellular redox regulation, was also markedly increased, reaching a 2.8-fold elevation compared with control levels (*p* < 0.001) (Figure 6B).

In Y79 cells, combination treatment was associated with a substantial reduction in the expression of the anti-apoptotic gene BCL-2, corresponding to a 58 ± 6% decrease relative to the control group (0.42-fold; *p* < 0.01). At the same time, CASP3 mRNA levels exhibited a pronounced increase, reaching a 3.6-fold elevation following combination therapy, which differed significantly from the effects observed with either single-agent treatment (*p* < 0.01).

**Figure 7:**
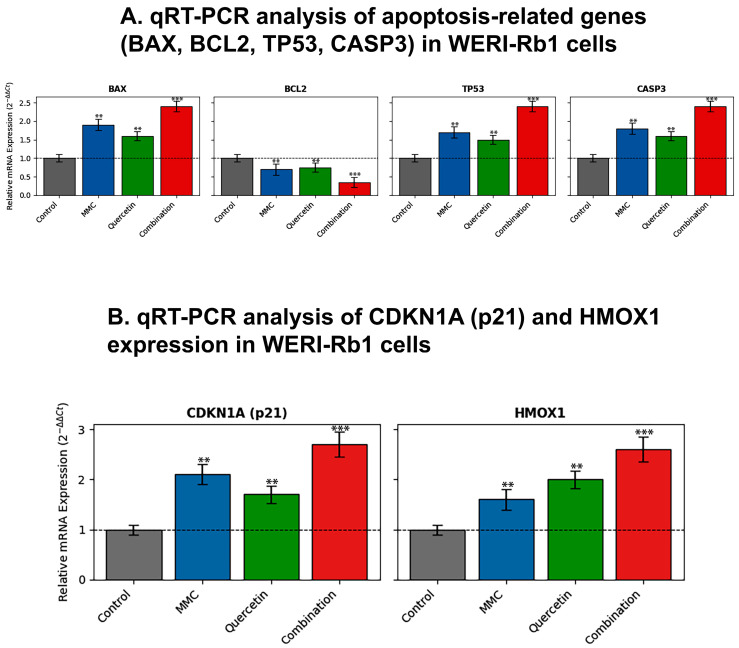
qRT-PCR analysis of apoptosis-, cell cycle-, and stress-related gene expression in WERI-Rb1 retinoblastoma cells following MMC and quercetin treatment. (**A**) Relative mRNA expression levels of the apoptosis-related genes BAX, BCL2, TP53, and CASP3 in WERI-Rb1 cells were evaluated by quantitative real-time polymerase chain reaction (qRT-PCR), normalized to GAPDH, and reported as fold changes relative to the control group. Compared with control conditions and single-agent treatments, combination treatment with MMC and quercetin led to a significant reduction in the expression of the anti-apoptotic gene BCL2, accompanied by pronounced increases in the expression of the pro-apoptotic genes BAX, TP53, and CASP3. (**B**) Relative mRNA expression levels of CDKN1A (p21) and HMOX1 in WERI-Rb1 cells were assessed by quantitative real-time polymerase chain reaction (qRT-PCR), normalized to GAPDH, and reported as fold changes relative to the control group. Compared with control conditions and single-agent treatments, combination treatment with MMC and quercetin yielded significantly higher expression levels of both CDKN1A and HMOX1. Data are presented as mean ± SD from three independent experiments. ** *p* < 0.01 vs. control; *** *p* < 0.001 vs. control and corresponding single-agent treatments.

A comparable transcriptional response was detected in WERI-Rb1 cells. Exposure to the combined treatment led to significant elevations in the expression of the pro-apoptotic genes BAX (2.6-fold), TP53 (2.9-fold), and CASP3 (3.1-fold), while concurrently reducing BCL-2 expression to 0.47-fold relative to control levels (*p* < 0.01) (Figure 7A).

In WERI-Rb1 cells, combination treatment induced a significant increase in CDKN1A (p21) expression, with transcript levels rising to 2.7-fold relative to control cells (*p* < 0.001). In parallel, expression of HMOX1 was strongly elevated, reaching a 2.6-fold increase compared with control levels (*p* < 0.001) (Figure 7B).

### 2.8. In Silico Protein–Protein Interaction and Pathway Analysis

**Figure 8:**
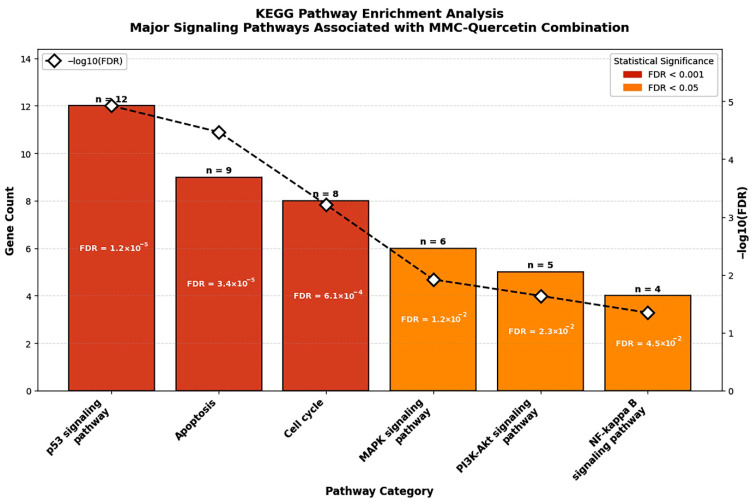
KEGG Pathway Enrichment Analysis of Molecular Pathways Associated with the MMC–Quercetin Combination. KEGG pathway enrichment analysis was conducted using STRING-derived protein–protein interaction data to identify molecular pathways associated with the combined effects of MMC and quercetin. Significantly enriched pathways were determined based on false discovery rate (FDR) values. Among the most prominently enriched categories were the p53 signaling pathway, apoptosis, and cell cycle-related pathways. Bar plots depict the number of genes mapped to each pathway, whereas the dashed line indicates −log10(FDR) values. Pathways with FDR < 0.05 were considered statistically significant. These analyses were performed to support mechanistic interpretation and do not constitute direct experimental validation.

**Figure 9:**
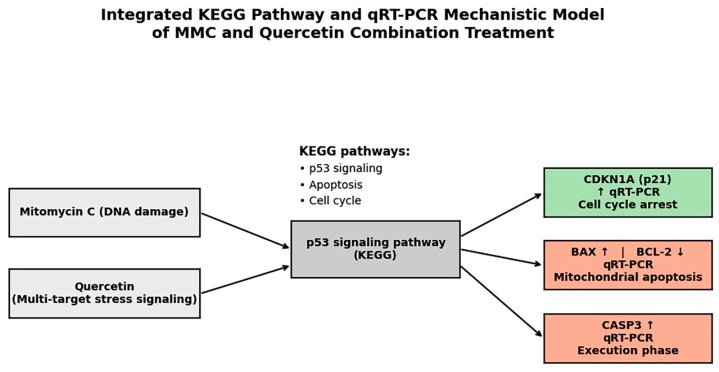
Schematic Representation of a Proposed Working Model Based on Transcriptional and Bioinformatic Analyses. A schematic model illustrating a hypothesis-generating working model underlying the synergistic effects of MMC and quercetin is presented. Based on KEGG pathway enrichment analysis and experimental qRT-PCR data, combination treatment is associated with transcriptional changes consistent with p53-related signaling pathways, thereby contributing to coordinated regulation of cell cycle arrest and apoptotic responses. Upregulation of CDKN1A (p21) is associated with induction of cell cycle arrest, whereas increased expression of BAX and CASP3, together with suppression of BCL-2, suggests transcriptional patterns compatible with mitochondrial apoptotic signaling. This model integrates in silico pathway analyses with experimentally observed transcriptional changes and should be interpreted as a conceptual framework rather than a direct experimental validation of signaling mechanisms.

Protein–protein interaction (PPI) network construction and pathway enrichment analyses were conducted using the STRING database to investigate molecular relationships underlying the combined effects of MMC and quercetin. This analysis demonstrated extensive functional interconnectivity among proteins associated with DNA damage responses, apoptotic regulation, and cell cycle control, with notable enrichment of pathways related to p53 signaling, apoptosis, and cell cycle regulation (Figure 8 and Figure 9).

**Figure 10:**
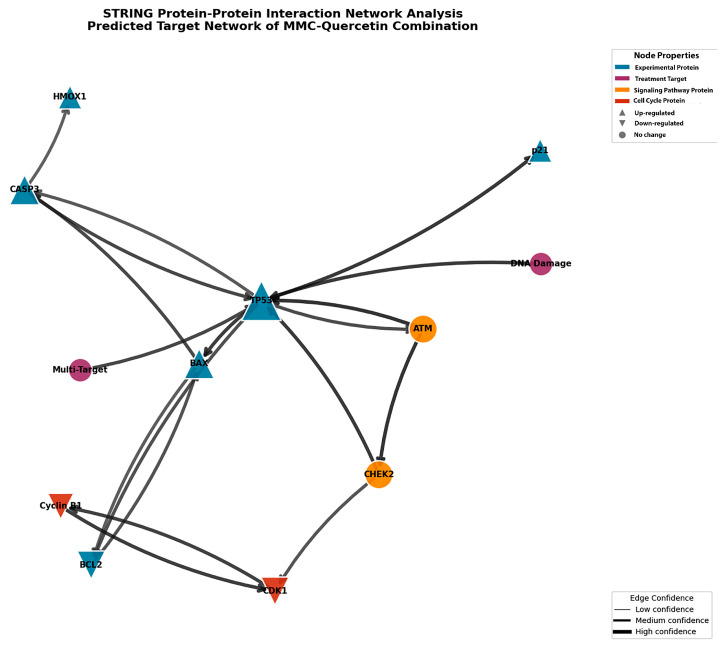
A protein–protein interaction network was constructed using the STRING database to visualize functional associations among proteins implicated in the effects of MMC and quercetin. The network comprises apoptosis- and cell cycle-related proteins, including TP53, BAX, BCL2, CASP3, CDKN1A, and HMOX1, and depicts their interaction patterns based on high-confidence STRING interaction scores. Within this network, TP53 displays the highest level of connectivity, underscoring its central role in coordinating the associated signaling interactions.

STRING network mapping revealed high-confidence interactions (confidence score > 0.7) among key regulatory proteins, including TP53, BAX, BCL-2, CASP3, CDKN1A, and HMOX1, highlighting a densely interconnected signaling network linking DNA damage sensing, mitochondrial apoptotic mechanisms, and cell cycle checkpoint control (Figure 10). Within this network, TP53 displayed the highest degree of connectivity, supporting its central role in orchestrating cellular stress responses and apoptotic signaling cascades.

In agreement with these interaction profiles, KEGG pathway enrichment analysis identified significant overrepresentation of the p53 signaling pathway (FDR = 1.2 × 10^−5^), apoptosis (FDR = 3.4 × 10^−5^), and cell cycle-associated pathways (FDR = 6.1 × 10^−4^), all of which correspond to molecular processes examined experimentally in the present study (Figure 8).

To integrate the in silico pathway associations with experimental observations, a schematic hypothesis-generating model was developed based on KEGG enrichment results and qRT-PCR findings (Figure 9). This model illustrates that the MMC–quercetin combination may be associated with p53-related signaling pathways, thereby promoting coordinated regulation of cell cycle arrest and apoptotic responses. Specifically, upregulation of CDKN1A (p21) is associated with enforcement of cell cycle arrest, whereas increased expression of BAX and CASP3 together with suppression of BCL-2 is consistent with activation of the intrinsic mitochondrial apoptotic pathway.

### 2.9. Effects of MMC and Quercetin in a 3D Retinoblastoma Tumor Spheroid Model

#### 2.9.1. Formation and Morphological Alterations of 3D Retinoblastoma Tumor Spheroids

Under control conditions, retinoblastoma cells formed compact, spherical, and well-organized three-dimensional tumor spheroids with smooth and regular borders. Treatment with quercetin alone resulted in a moderate reduction in spheroid size while largely preserving overall spheroid integrity. In contrast, MMC treatment induced a more pronounced decrease in spheroid size, accompanied by partial loss of spheroid compactness.

**Figure 11:**
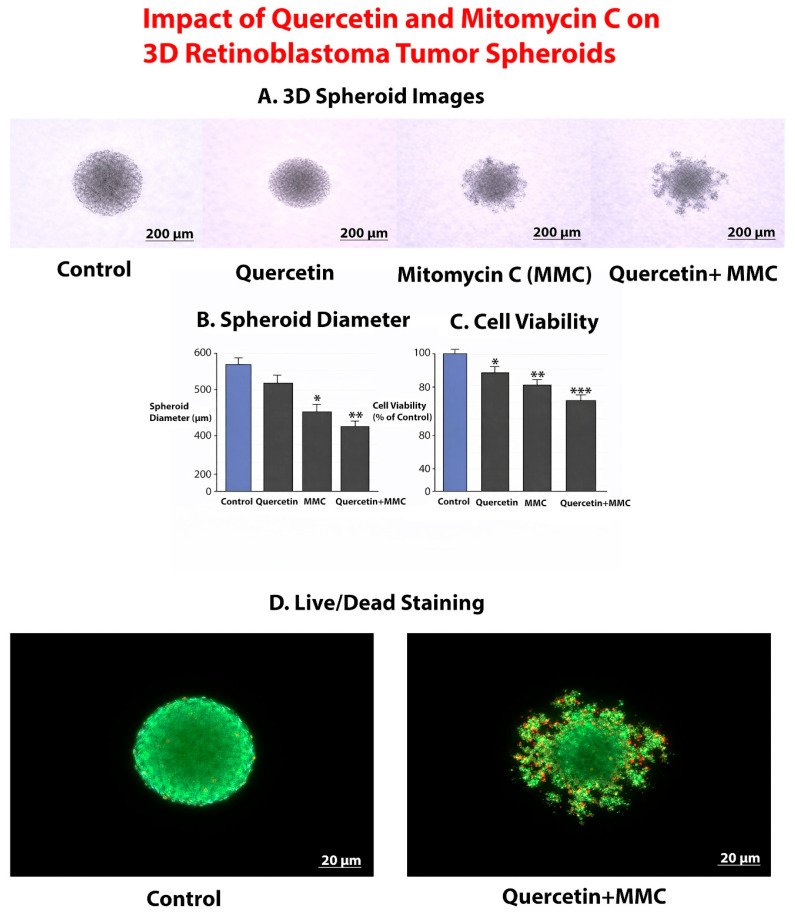
Quercetin enhances MMC-induced cytotoxicity in 3D retinoblastoma tumor spheroids. (**A**) Representative bright-field images of three-dimensional retinoblastoma tumor spheroids under control conditions and following treatment with quercetin, MMC, or their combination. Control spheroids exhibit a compact and spherical morphology, whereas MMC treatment induces spheroid shrinkage and partial loss of structural integrity. Combined quercetin and MMC treatment results in pronounced disruption of spheroid architecture with irregular borders and reduced compactness. Scale bar = 200 µm. (**B**) Spheroid diameter (µm) measured as the mean of two perpendicular axes. MMC treatment significantly reduced spheroid diameter compared with control (* *p* < 0.05), while the most pronounced reduction was observed following combined quercetin and MMC treatment (** *p* < 0.01). (**C**) Cell viability (% of control) was assessed using an ATP-based luminescence assay optimized for three-dimensional cultures. Quercetin, MMC, and quercetin + MMC treatments resulted in a stepwise reduction in spheroid viability, with the greatest decrease observed in the combination group (* *p* < 0.05, ** *p* < 0.01, *** *p* < 0.001 versus control). (**D**) Representative live/dead fluorescence staining of 3D tumor spheroids. Control spheroids display predominant green fluorescence, indicating high cell viability, whereas quercetin + MMC-treated spheroids show marked disruption of spheroid integrity accompanied by increased red fluorescence, consistent with enhanced cell death. Scale bar = 20 µm.

Notably, combined MMC and quercetin treatment led to marked spheroid shrinkage and substantial disruption of spheroid architecture, characterized by irregular borders, reduced cellular density, and partial fragmentation (Figure 11A).

#### 2.9.2. Quantitative Reduction in Spheroid Size Following Combination Treatment

Quantitative analysis of spheroid diameter revealed clear differences among treatment groups. Compared with control spheroids, both MMC and quercetin treatments significantly reduced spheroid diameter. The most pronounced reduction was observed in the MMC + quercetin group, which exhibited significantly smaller spheroids than those treated with either agent alone (Figure 11B). These findings indicate that the antiproliferative effects observed in two-dimensional cultures are preserved and further enhanced in a three-dimensional tumor spheroid model.

#### 2.9.3. Suppression of Cell Viability in 3D Tumor Spheroids

Cell viability assessment of 3D tumor spheroids demonstrated that treatment with MMC or quercetin alone significantly reduced spheroid viability compared with vehicle-treated control spheroids. Importantly, combined MMC and quercetin treatment resulted in the strongest suppression of spheroid viability, exceeding the effects observed with either single-agent treatment (Figure 11C). These results indicate that the synergistic cytotoxic interaction between MMC and quercetin identified in monolayer cultures is maintained within the 3D spheroid context.

#### 2.9.4. Live/Dead Fluorescence Patterns Following Combination Treatment

Live/dead fluorescence staining revealed predominantly green fluorescence in control spheroids, indicating a high proportion of viable cells. Spheroids treated with either MMC or quercetin alone exhibited increased red fluorescence, consistent with treatment-induced cell death. In contrast, spheroids exposed to the MMC + quercetin combination displayed extensive red fluorescence accompanied by a marked reduction in green signal, reflecting widespread loss of cell viability and pronounced disruption of spheroid architecture (Figure 11D).

### 2.10. NAC Pretreatment Partially Attenuates MMC- and Quercetin-Induced ROS Generation and Cytotoxicity in 3D Retinoblastoma Tumor Spheroids

Treatment of three-dimensional retinoblastoma tumor spheroids with MMC resulted in a marked increase in intracellular ROS levels compared with control spheroids, consistent with MMC-induced DNA damage and oxidative stress-mediated cytotoxicity. Quercetin treatment alone induced a modest alteration in intracellular ROS levels relative to control spheroids, remaining substantially lower than the levels observed following MMC treatment alone.

**Figure 12:**
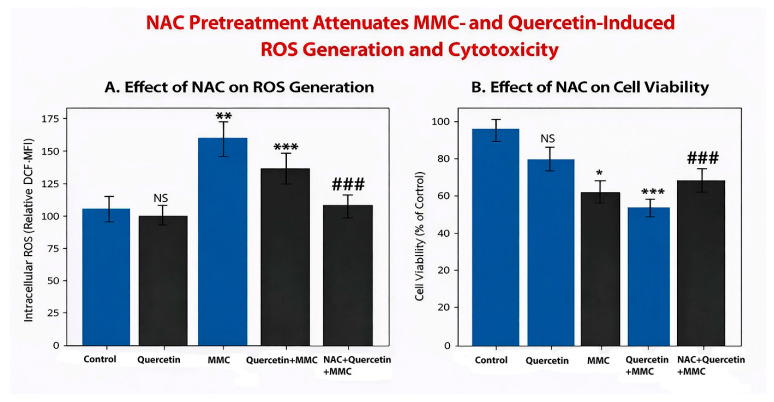
NAC pretreatment attenuates MMC- and quercetin-induced ROS generation and cytotoxicity in 3D retinoblastoma tumor spheroids. (**A**) Intracellular ROS levels in 3D retinoblastoma tumor spheroids following treatment with quercetin, MMC, or their combination, with or without N-acetyl-L-cysteine (NAC) pretreatment. ROS levels were quantified using DCFH-DA fluorescence and expressed as relative mean fluorescence intensity (DCF-MFI) normalized to control spheroids. MMC treatment significantly increased intracellular ROS levels (** *p* < 0.01 vs. control), and combined quercetin and MMC treatment also resulted in significantly elevated ROS levels compared with control spheroids (*** *p* < 0.001 vs. control). Quercetin alone did not significantly alter basal ROS levels (NS vs. control). NAC pretreatment markedly reduced ROS accumulation in quercetin + MMC-treated spheroids (### *p* < 0.001 vs. quercetin + MMC). (**B**) Cell viability (% of control) of 3D retinoblastoma tumor spheroids following the indicated treatments, assessed using an ATP-based luminescence assay. Quercetin treatment did not significantly affect spheroid viability compared with control (NS). MMC treatment significantly reduced cell viability (* *p* < 0.05 vs. control), whereas combined quercetin and MMC treatment induced a more pronounced cytotoxic effect (*** *p* < 0.001 vs. control). NAC pretreatment partially restored cell viability in the quercetin + MMC group (### *p* < 0.001 vs. quercetin + MMC), without fully reversing cytotoxicity. Data are presented as mean ± SD from at least three independent experiments.

In spheroids treated with the MMC + quercetin combination, intracellular ROS levels were significantly elevated compared with control spheroids but were lower than those induced by MMC alone, indicating a modulatory effect of quercetin on MMC-induced oxidative stress. Importantly, pretreatment with the ROS scavenger N-acetyl-L-cysteine (NAC) significantly reduced intracellular ROS accumulation in combination-treated spheroids, as evidenced by a marked decrease in DCF fluorescence intensity. NAC treatment alone did not significantly alter basal ROS levels compared with control spheroids (Figure 12A).

#### 2.10.1. Partial Reversal of MMC + Quercetin-Induced Cytotoxicity by ROS Scavenging

Consistent with the reduction in intracellular ROS levels, NAC pretreatment significantly attenuated the cytotoxic effects induced by combined MMC and quercetin treatment in three-dimensional retinoblastoma tumor spheroids. Spheroids pretreated with NAC exhibited significantly higher cell viability compared with spheroids treated with the MMC + quercetin combination alone (Figure 12B).

However, NAC pretreatment did not fully restore cell viability to control levels, indicating that ROS scavenging provides partial, but incomplete, protection against MMC + quercetin-induced cytotoxicity under three-dimensional culture conditions.

#### 2.10.2. ROS Contributes to, but Does Not Fully Mediate, MMC + Quercetin-Induced Cell Death

Although NAC-mediated neutralization of ROS significantly reduced oxidative stress and partially restored spheroid viability, the incomplete reversal of cytotoxicity indicates that ROS generation contributes to, but does not solely account for, MMC + quercetin-induced cell death. These findings suggest that the synergistic cytotoxic effects of the combination in three-dimensional retinoblastoma tumor spheroids involve ROS-dependent mechanisms acting in concert with additional ROS-independent pathways, including mitochondrial apoptotic signaling and cell cycle dysregulation.

Collectively, these results support a multifactorial mode of action underlying the enhanced cytotoxic response observed following combined MMC and quercetin treatment in three-dimensional retinoblastoma tumor spheroids.

## 3. Discussion

In the present study, combined treatment with MMC and quercetin produced enhanced antitumor activity in retinoblastoma cell models compared with either agent alone. This effect was supported by integrated in vitro and in silico analyses demonstrating synergistic cytotoxicity, induction of G2/M cell cycle arrest, increased apoptotic response, suppression of pro-angiogenic and pro-inflammatory mediators, and coordinated regulation of genes involved in apoptosis and cell cycle control. Dose–response modeling and combination index analyses consistently indicated synergistic interactions across multiple effect levels, suggesting that effective cytotoxic responses may be achieved at lower drug concentrations. Mechanistically, the observed synergy was associated with G2/M phase accumulation, expansion of apoptotic cell populations, transcriptional upregulation of pro-apoptotic and cell cycle regulatory genes, downregulation of anti-apoptotic signaling, and modulation of p53-associated molecular networks. These findings were further supported by PPI and KEGG pathway analyses, which highlighted interconnected signaling pathways linking DNA damage responses, mitochondrial apoptosis, and cell cycle regulation. Overall, the data support a model in which quercetin enhances MMC-induced antitumor activity through coordinated effects on cell cycle arrest, apoptosis-related transcriptional regulation, and ROS-associated cellular stress. This integrated framework provides a biological rationale for combination-based strategies aimed at improving therapeutic efficacy while potentially reducing MMC-associated toxicity in retinoblastoma.

Recent advances in flavonoid-based anticancer research indicate that these compounds function as multi-target modulators that regulate tumor cell fate through coordinated control of interconnected cellular processes rather than isolated signaling pathways [18,19]. Mechanistically, structurally related flavonoids, including quercetin, have been shown to influence apoptosis through integrated modulation of mitochondrial signaling, intracellular redox balance, and transcriptional regulation, including p53-associated pathways [20]. Regulation of key signaling axes such as PI3K AKT, MAPK, NF κB, and JAK STAT further supports the capacity of quercetin to reshape cellular responses to stress and therapeutic intervention. In this context, modulation of reactive oxygen species represents a critical component of this network, where redox dynamics interact with transcriptional and apoptotic signaling to influence cell survival and death decisions [21,22]. In addition, emerging evidence indicates that flavonoids may enhance chemotherapeutic efficacy not only through signaling-based mechanisms but also by influencing intracellular drug behavior, including cellular uptake, retention, and bioavailability. These findings support a systems-level model in which flavonoids act as integrative regulators linking redox homeostasis, transcriptional control, mitochondrial apoptosis, and intracellular drug dynamics. Within this framework, the enhanced cytotoxicity observed following combined MMC and quercetin treatment may reflect the convergence of these mechanisms, whereby quercetin potentiates MMC-induced stress responses and reinforces overall therapeutic effectiveness [19].

Retinoblastoma continues to represent the most frequently diagnosed intraocular malignancy in childhood, and despite advances in multimodal therapeutic approaches, metastatic progression and the development of treatment resistance remain major clinical challenges [23]. Although MMC is commonly utilized as a topical adjuvant agent, particularly in the management of ocular surface neoplasms, its broader clinical applicability is limited by dose-dependent ocular toxicities, including scleral thinning and conjunctival ischemia, as well as the emergence of tumor cell resistance mechanisms [24,25]. In light of these constraints, the current investigation examined whether quercetin, a naturally occurring flavonoid with established anticancer activity, could potentiate the antitumor effects of MMC in retinoblastoma cells while providing mechanistic insight into this interaction. The data indicate that combined administration of low-dose MMC and quercetin elicits pronounced synergistic cytotoxicity together with robust induction of apoptotic cell death in both Y79 and WERI-Rb1.

Synergistic interactions between MMC and quercetin were quantitatively validated using the Chou–Talalay approach, with CI values consistently remaining below unity (0.58–0.81), supporting the presence of true pharmacological synergy rather than simple additivity [26]. From a mechanistic perspective, this interaction is plausibly attributable to the complementary biological actions of the two compounds. The cytotoxic activity of MMC is predominantly mediated through alkylation of guanine residues, leading to DNA cross-link formation and subsequent activation of DNA damage response pathways [27]. By contrast, quercetin has been shown to interfere with cellular adaptive mechanisms that normally mitigate such damage, through modulation of multiple survival- and stress-associated signaling networks. Previous investigations have demonstrated that quercetin suppresses key oncogenic pathways, including PI3K/AKT/mTOR, MAPK/ERK, and NF-κB, which play central roles in cancer cell survival, proliferation, and therapeutic resistance [28,29]. In line with these observations, STRING-based protein–protein interaction analysis in the present work suggests that quercetin-associated pathway modulation may amplify MMC-induced DNA damage signaling by reducing the apoptotic threshold, particularly within p53-centered regulatory networks. In addition, quercetin has been reported to inhibit drug efflux transporters such as P-glycoprotein, a mechanism that could further enhance synergistic efficacy by increasing intracellular accumulation and retention of MMC [30]. Collectively, these mechanistic considerations provide a plausible framework to explain the enhanced antitumor activity observed with the MMC–quercetin combination.

From a DNA damage response perspective, the observed synergy can be interpreted in the context of altered cellular recovery mechanisms following MMC-induced genotoxic stress. MMC exerts its cytotoxic activity primarily through DNA cross-link formation, which activates DNA damage response (DDR) pathways and cell cycle checkpoint signaling. In this setting, quercetin may enhance cytotoxic efficacy by interfering with cellular adaptation to DNA damage, potentially through modulation of DNA repair processes, attenuation of checkpoint recovery, or prolongation of DNA damage signaling. Such effects could lead to persistence of DNA lesions and sustained activation of stress-responsive pathways, thereby shifting the cellular outcome from transient cell cycle arrest toward irreversible apoptotic commitment. Although direct assessment of DNA damage markers (e.g., γH2AX foci formation) or repair pathway components was not performed in the present study, the combined transcriptional activation of p53-associated pathways together with enhanced apoptotic response is consistent with a model in which quercetin potentiates MMC-induced DNA damage signaling by limiting cellular recovery capacity.

A prominent observation arising from this study is that combination treatment is associated with a pronounced apoptotic response that displays coherence across both cellular and molecular levels. Flow cytometric evaluation based on Annexin V/PI staining revealed that exposure to MMC and quercetin in combination led to an approximate twofold increase in apoptotic cell populations relative to single-agent treatments in both Y79 and WERI-Rb1 cell lines (Figure 3B and Figure 4). This cellular response was paralleled by transcriptional alterations, including increased expression of the pro-apoptotic gene BAX and the tumor suppressor TP53, together with a concomitant reduction in expression of the anti-apoptotic gene BCL-2 (Figure 6 and Figure 7). The consequent alteration in the BAX/BCL-2 expression balance represents a critical regulatory event in mitochondrial outer membrane permeabilization, facilitating cytochrome c release and downstream activation of the caspase cascade [31]. Collectively, these observations support the interpretation that the synergistic cytotoxic activity of the MMC–quercetin combination is associated with engagement of the intrinsic mitochondrial apoptotic pathway. However, apoptosis assessment in the present study is primarily based on Annexin V/PI staining and transcriptional profiling, without direct validation of execution-phase events such as caspase activation or PARP cleavage. Therefore, these findings should be interpreted as indicative of apoptotic processes rather than definitive evidence of apoptosis.

The observed induction of pro-apoptotic gene expression following combination treatment supports the involvement of p53-dependent apoptotic signaling as a central contributor to the synergistic interaction between MMC and quercetin. This interpretation is grounded in the well-established capacity of MMC to activate p53 signaling pathways as a consequence of DNA damage [32], together with prior evidence demonstrating that quercetin can promote p53 stabilization and enhance its transcriptional activity, at least partially through interference with MDM2-mediated degradation processes [33]. In this context, coordinated modulation of p53-regulated apoptotic gene expression is consistent with a potential transcriptional basis for the enhanced cytotoxic efficacy associated with the MMC–quercetin combination.

It should be emphasized, however, that the present findings are primarily based on transcriptional analyses and do not include direct functional interrogation of p53 dependency. Although concurrent upregulation of TP53 and CDKN1A was observed, the study does not provide direct evidence that CDKN1A induction is transcriptionally mediated by p53, nor does it establish a causal role for p53 in mediating the observed synergistic effects. Therefore, the relationship between TP53 expression and downstream gene activation should be interpreted as associative rather than causally established. Definitive confirmation of p53-dependent transcriptional regulation would require promoter-level analyses or loss-of-function approaches, such as p53 knockdown or pharmacological inhibition, which were beyond the scope of the current study. Accordingly, the involvement of p53 signaling in the MMC–quercetin interaction should be considered mechanistically suggestive but not conclusive. Importantly, these observations are limited to transcriptional associations and do not establish functional activation of p53 signaling at the protein level.

Beyond the induction of apoptosis, reinforcement of G2/M phase cell cycle arrest appears to represent an additional mechanism contributing to the antitumor activity observed with the MMC–quercetin combination. MMC has been well documented to disrupt DNA replication and repair through DNA cross-link formation, leading to accumulation of cells in the S and G2 phases of the cell cycle [34]. In the current experimental setting, co-administration of quercetin was associated with a further intensification of G2/M arrest, pointing to a cooperative influence on cell cycle checkpoint control mechanisms. This effect is closely linked to the pronounced induction of CDKN1A (p21) expression detected following combination treatment. As a key negative regulator of the cyclin B1/CDK1 complex, p21 serves a pivotal function in enforcing activation of the G2/M checkpoint [35]. Prolonged engagement of this checkpoint has been shown to shift cellular outcomes from reversible growth arrest toward commitment to programmed cell death, a transition that may partially explain the elevated levels of apoptosis detected under combination treatment conditions [36].

However, it should be noted that the current interpretation of G2/M arrest is primarily based on flow cytometric cell cycle distribution and transcriptional upregulation of CDKN1A, and does not include direct evaluation of key checkpoint regulators such as CDK1 phosphorylation status, Cyclin B1 accumulation, or checkpoint kinase activation. Therefore, while the observed findings are consistent with activation of G2/M checkpoint control mechanisms, they do not provide definitive evidence of checkpoint engagement at the protein level. In the present study, increased CDKN1A (p21) expression provides indirect support for checkpoint involvement, given its established role in regulating CDK1/Cyclin B1 activity and G2/M transition [35,36]. Entry into mitosis is tightly controlled by activation of the Cyclin B–CDK1 complex, which is regulated through checkpoint pathways that preserve genomic integrity [37]. In response to DNA damage, these pathways suppress Cyclin B–CDK1 activation and prevent premature mitotic entry, thereby enforcing G2/M arrest [38,39]. In this context, the observed G2/M accumulation is biologically consistent with checkpoint-mediated inhibition of mitotic entry; however, direct confirmation of these regulatory events remains to be established.

In addition to apoptotic and cell cycle-related mechanisms, the involvement of oxidative stress appears to constitute an additional layer contributing to the cytotoxic activity of the combination treatment. Concurrent elevations in intracellular ROS levels together with increased expression of HMOX1 point toward engagement of stress-responsive pathways linked to redox imbalance. Quercetin has been reported to display context-dependent antioxidant or pro-oxidant properties, with its biological effects varying according to concentration and cellular environment [40]. Under the relatively high concentration conditions applied in this study, quercetin may facilitate ROS accumulation, potentially through redox cycling and interactions with intracellular metal ions, thereby intensifying MMC-associated oxidative stress [21]. An increase in oxidative burden of this nature is expected to aggravate DNA damage and impair mitochondrial function, processes that can lower the threshold for apoptotic activation. Such effects provide a plausible mechanistic explanation for the reinforcement of cytotoxic synergy observed upon combined exposure to MMC and quercetin.

Notably, the observation that MMC alone induces higher ROS levels, whereas the combination treatment results in greater cytotoxicity, indicates that ROS generation is not the sole determinant of cell death in this system. Instead, ROS appears to function as a modulatory component that interacts with parallel mechanisms, including DNA damage signaling, cell cycle checkpoint regulation, and apoptotic pathway activation. In this context, the enhanced cytotoxicity observed under combination treatment is more likely attributable to the convergence of these mechanisms rather than to absolute ROS levels alone. Furthermore, the partial attenuation of cytotoxic effects following NAC pretreatment supports a contributory, but not exclusive, role for oxidative stress. Although quantitative redox profiling and pathway-specific analyses were not performed in the present study, the available data are consistent with a model in which ROS acts as an amplifier of MMC-induced cytotoxic signaling rather than as a primary driver.

**Figure 13:**
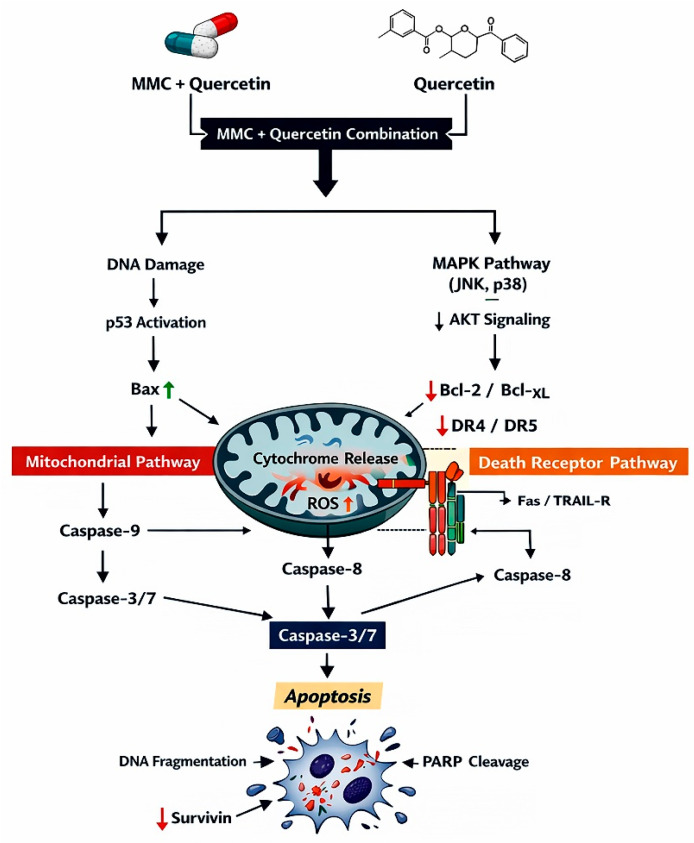
Proposed Working Model Illustrating the Synergistic Antitumor Effects of Combined MMC and Quercetin Treatment in Retinoblastoma Cells. A schematic model is presented to illustrate a conceptual framework describing the synergistic antitumor effects observed following combined treatment with MMC and quercetin in retinoblastoma cells. Based on the experimental findings generated in this study, DNA damage induced by MMC is linked to transcriptional responses consistent with p53-associated signaling pathways, accompanied by increased expression of pro-apoptotic mediators, including BAX and CASP3, together with suppression of the anti-apoptotic protein BCL-2, thereby suggesting engagement of mitochondrial apoptotic signaling. In this context, quercetin may enhance cellular sensitivity to MMC-induced stress, resulting in amplified p53-associated transcriptional responses, CDKN1A (p21)-mediated cell cycle arrest, and reinforcement of apoptotic signaling pathways. Elevated oxidative stress, supported by HMOX1 upregulation and partial attenuation of cytotoxic effects following NAC pretreatment, contributes to but does not fully explain the observed cytotoxicity. This schematic is intended to represent a data-driven working model integrating qRT-PCR, flow cytometry, ELISA, and in silico analyses, rather than direct experimental validation of all signaling interactions.

The experimental evidence generated in this study is consistent with a mechanistic framework in which MMC and quercetin act on convergent p53-associated apoptotic signaling pathways to produce synergistic cytotoxic effects in retinoblastoma cells. As depicted in Figure 13, DNA damage induced by MMC is linked to engagement of p53-centered regulatory networks at the transcriptional level, accompanied by increased expression of pro-apoptotic mediators, including BAX and CASP3, together with suppression of the anti-apoptotic protein BCL-2, thereby favoring commitment to the mitochondrial apoptotic program. In this context, quercetin appears to amplify these effects by reducing the apoptotic threshold through modulation of multiple stress-responsive pathways, ultimately resulting in enhanced p53-dependent transcriptional responses.

The proposed model incorporates both transcriptional and functional evidence generated in the present study, including enhanced apoptotic cell death, an increased BAX/BCL-2 expression ratio, increased CASP3 expression consistent with apoptotic signaling, and CDKN1A (p21)-associated cell cycle arrest. Although oxidative stress contributes to the observed cytotoxic effects, as indicated by HMOX1 upregulation and the partial attenuation of cytotoxicity following NAC pretreatment, the lack of complete rescue suggests that apoptotic induction is not solely dependent on ROS generation. Accordingly, Figure 13 is intended to be viewed as a data-driven working model that illustrates how DNA damage signaling, mitochondrial apoptotic pathways, and cell cycle checkpoint activation act in concert to underlie the synergistic antitumor activity of the MMC–quercetin combination, rather than as an exhaustive representation of all potential signaling interactions.

However, it should be acknowledged that although the three-dimensional spheroid model employed in this study provides both morphological and functional evidence of treatment response, including reductions in spheroid size, decreased overall viability, and spatially resolved live and dead staining patterns, the analysis remains primarily phenotypic. The heterogeneous distribution of dead cells suggests that the MMC and quercetin combination affects both peripheral and inner spheroid regions, indicating partial overcoming of diffusion-related barriers. However, these observations are not supported by direct quantitative assessment of drug penetration, spatial viability gradients, or microenvironment-driven resistance mechanisms. Therefore, while the current findings support enhanced efficacy under 3D conditions, further studies incorporating quantitative and spatially resolved analyses will be required to definitively establish the capacity of this combination to overcome intrinsic 3D tumor resistance features.

Incorporation of tumor microenvironment-related parameters into the analysis offers additional insight into the potential anti-angiogenic and immunomodulatory properties of the MMC–quercetin combination. Within this framework, the marked reduction in secretion of vascular endothelial growth factor (VEGF) and interleukin-6 (IL-6) constitutes a notable observation. VEGF is widely recognized as a central driver of angiogenesis and tumor maintenance across multiple malignancies, including retinoblastoma, where it facilitates pathological neovascularization and disease progression [41]. IL-6 similarly contributes to tumor development by supporting cancer cell survival, proliferation, and immune evasion through persistent inflammatory signaling within the tumor microenvironment [42]. In the present work, assessment of VEGF and IL-6 secretion was confined to Y79 cells, as this cell line displayed a more consistent and reproducible secretory response under the experimental conditions employed.

The reduction in VEGF and IL-6 secretion observed in this study aligns with established molecular activities attributed to quercetin. Prior investigations have indicated that quercetin can limit VEGF expression by interfering with stabilization of hypoxia-inducible factor-1α (HIF-1α) [43] and can reduce IL-6 production through suppression of NF-κB-dependent transcriptional signaling [44]. Such mechanisms imply that, in addition to direct cytotoxic effects, the MMC–quercetin combination may exert supplementary antitumor activity by perturbing pro-angiogenic and pro-inflammatory signaling pathways that contribute to tumor persistence and progression. This consideration is particularly pertinent in retinoblastoma, where modulation of angiogenic signaling within the intraocular environment has emerged as a therapeutic objective, and anti-VEGF approaches, including bevacizumab administration, are actively under clinical investigation as adjunctive treatment strategies [45]. In this context, the combined use of MMC and quercetin may offer therapeutic benefit not only through the induction of tumor cell death but also by adversely influencing components of the tumor-supportive microenvironment.

To reduce dependence on a single experimental system, two phenotypically and genetically distinct retinoblastoma cell lines were incorporated into the experimental design, thereby strengthening the robustness of the observations. Y79 and WERI-Rb1 cells differ in their molecular background and biological behavior, offering complementary platforms for assessment of therapeutic responsiveness [46]. The detection of synergistic interactions between MMC and quercetin in both cellular models indicates that the observed effects are not confined to a single retinoblastoma context, but may instead reflect a broader applicability within this tumor type. Despite this consistency, additional validation using a wider spectrum of ocular cancer cell lines remains necessary. In particular, extension of the analysis to other malignancies, such as uveal melanoma (e.g., Mel270, OMM2.3) or conjunctival carcinoma, would be required to determine whether the synergistic interaction observed here is maintained beyond retinoblastoma.

Careful consideration of study limitations is required for the appropriate interpretation of these findings. A major constraint of the current work is its reliance on in vitro experimental systems, which do not fully recapitulate key features of the in vivo tumor microenvironment, including three-dimensional tissue architecture, cell–matrix interactions, pharmacokinetic behavior, and systemic immune influences [47]. In addition, the drug concentrations and exposure durations applied under controlled experimental conditions may not accurately reflect those achieved during topical or intraocular administration of MMC in clinical settings [48]. Further limitations arise from the pharmacological characteristics of quercetin, particularly its limited systemic bioavailability and rapid metabolic clearance, although these constraints may be less pronounced in localized ocular applications [49]. An important translational limitation of the present study is the absence of selectivity analysis in non-malignant ocular cells. Although the MMC–quercetin combination demonstrated enhanced cytotoxic efficacy in retinoblastoma cell lines at reduced drug concentrations, comparative toxicity assessments in normal retinal or ocular cell models were not performed. As a result, it remains unclear whether the observed sensitization reflects tumor-specific effects or a generalized increase in cytotoxicity. Given the known dose-dependent ocular toxicity of MMC, evaluation of the therapeutic window through direct comparison with non-malignant systems is essential for future studies. Therefore, any implications regarding improved safety or selectivity should be interpreted with caution until tumor-specific selectivity is experimentally validated. Mechanistic interpretation of the findings is further limited by the reliance on transcriptional profiling. While gene expression changes provide insight into pathway engagement, they do not establish functional protein activity. Key apoptotic and cell cycle regulators, including BAX, BCL-2, CASP3, and CDKN1A, as well as post-translational events such as PARP cleavage, were not validated at the protein level. Accordingly, incorporation of protein-based analyses, such as Western blotting or activity assays, will be necessary in future studies to confirm the proposed molecular mechanisms.

Future investigations will be required to extend the findings of this study across several complementary directions. Evaluation of the MMC–quercetin combination in vivo, particularly using retinoblastoma xenograft mouse models, would be critical for determining translational relevance and therapeutic efficacy within a physiologically representative context [21]. In parallel, deeper mechanistic characterization through high-throughput methodologies, including transcriptomic profiling (RNA-seq) or kinase activity-based analyses, may facilitate identification of key molecular nodes responsible for the observed synergistic interaction. Additional forms of regulated cell death, such as DNA damage response signaling marked by γH2AX foci formation, as well as autophagy and ferroptosis, also warrant systematic exploration. Advancement toward clinical application will further depend on formulation-oriented strategies. Development of optimized ocular delivery systems, including nanoparticle-based carriers, liposomal formulations, or in situ gel platforms, may enhance quercetin stability, ocular penetration, and bioavailability, thereby improving its therapeutic performance [50,51]. Moreover, comprehensive preclinical safety assessments, incorporating both ex vivo and in vivo toxicity evaluations in corneal epithelium, retinal pigment epithelium, and other ocular tissues, will be essential to define the safety profile of this combination strategy prior to clinical translation [52]. Although transcriptional upregulation of TP53 and p53-regulated genes was observed, protein-level validation of p53-associated signaling was not performed. Therefore, the involvement of p53-associated signaling pathways should be interpreted as associative rather than direct evidence of functional p53-associated signaling.

## 4. Materials and Methods

### 4.1. Chemicals and Reagents

MMC and quercetin dihydrate (CAS No. 6151-25-3) were sourced from Sigma-Aldrich (St. Louis, MO, USA). For experimental use, MMC was formulated as a 2 mM stock solution in sterile distilled water, divided into single-use aliquots to avoid repeated freeze–thaw exposure, and stored at −20 °C under light-protected conditions. Quercetin was solubilized in dimethyl sulfoxide (DMSO; Sigma-Aldrich) to generate a 100 mM stock solution, which was likewise maintained at −20 °C until use.

Immediately prior to each experiment, working concentrations were prepared by diluting stock solutions in complete cell culture medium. The final concentration of DMSO in all treatment conditions was kept at or below 0.1% (*v*/*v*), a level verified to exert no detectable effect on cell viability. Vehicle control groups were established using equivalent concentrations of DMSO.

RPMI-1640 medium, fetal bovine serum (FBS), penicillin–streptomycin, and trypsin–EDTA were obtained from Gibco (Thermo Fisher Scientific, Waltham, MA, USA). Cell viability measurements were carried out using the Cell Counting Kit-8 (CCK-8; Sigma-Aldrich), whereas apoptotic cell death was evaluated using an Annexin V-FITC/PI apoptosis detection kit (BD Biosciences, San Jose, CA, USA). All reagents were of analytical grade and handled in accordance with manufacturer-provided specifications.

### 4.2. Culture Conditions and Maintenance of Human Retinoblastoma Cell Lines

Human retinoblastoma cell lines Y79 (ATCC^®^ HTB-18™) and WERI-Rb1 (ATCC^®^ HTB-169™) were obtained from the American Type Culture Collection (Manassas, VA, USA). Following receipt, both cell lines were expanded under controlled culture conditions and subsequently cryopreserved to generate master and working cell stocks. All experimental procedures were conducted using cells maintained within a defined passage window in order to limit phenotypic drift and genetic alterations associated with extended in vitro cultivation.

Cells were propagated under semi-suspension growth conditions in RPMI-1640 medium supplemented with 10% heat-inactivated FBS and 1% penicillin–streptomycin solution (100 U/mL penicillin and 100 µg/mL streptomycin). Cultures were maintained at 37 °C in a humidified incubator with a controlled atmosphere of 5% CO_2_. Throughout routine maintenance, cell density, aggregation behavior, and morphological features were periodically assessed by phase-contrast microscopy to confirm stable growth characteristics and absence of microbial contamination.

To sustain appropriate nutrient availability and metabolic homeostasis, culture medium renewal was performed at 2–3 day intervals. Medium replacement involved gentle centrifugation followed by resuspension in fresh medium, a procedure selected to minimize mechanical stress and preserve cellular integrity. Cell density was carefully regulated during maintenance to avoid excessive aggregation, nutrient exhaustion, or hypoxia-related stress that could confound treatment responses.

For all experimental applications, cells were collected during the logarithmic growth phase, corresponding to approximately 70–80% effective confluence. Prior to experimental manipulation, cell number and viability were determined using hemocytometer-based counting in combination with trypan blue exclusion. Only cultures exhibiting viability exceeding 95% were advanced to downstream analyses to ensure consistency and reproducibility.

This standardized culture workflow was applied uniformly across all experimental assays performed in the study, including two-dimensional cytotoxicity measurements, apoptosis analyses, gene expression experiments, and three-dimensional tumor spheroid generation, thereby ensuring methodological consistency across experimental platforms.

### 4.3. Assessment of Cell Viability and Proliferative Capacity Using the CCK-8 Assay

Cell viability and proliferative activity were quantified using the CCK-8 assay. Y79 and WERI-Rb1 cells were dispensed into 96-well flat-bottom plates at a density of 5 × 10^3^ cells per well in complete RPMI-1640 medium. Following plating, a 24 h equilibration period was allowed under standard culture conditions prior to initiation of drug exposure.

Subsequently, cells were exposed to increasing concentrations of MMC (0.1, 0.5, 1, 5, and 10 µM) and quercetin (5, 10, 25, 50, and 100 µM), administered either individually or as fixed-ratio combinations. Treatments were maintained for 24 or 48 h to evaluate both early and time-dependent effects on cell viability. Each experimental condition was assessed in a minimum of triplicate wells, and all experiments were reproduced at least three times independently.

Upon completion of the treatment interval, CCK-8 reagent (10 µL) was introduced directly into each well containing 100 µL of culture medium. Plates were returned to a humidified incubator at 37 °C for an additional 2 h to allow development of the colorimetric reaction. Formazan production, reflecting the number of metabolically active cells, was determined by measuring absorbance at 450 nm using a microplate reader.

Viability values were calculated as percentages relative to untreated control cultures. Results are reported as mean ± standard deviation derived from a minimum of three independent experiments. Concentration–response relationships were established, and half-maximal inhibitory concentration (IC_50_) values were derived by nonlinear regression analysis using GraphPad Prism version 9.0 (GraphPad Software, San Diego, CA, USA).

Based on the IC_50_ values obtained from single-agent dose–response experiments, MMC and quercetin were subsequently used at concentrations corresponding to their respective IC_50_ values in mechanistic assays. In Y79 cells, these concentrations corresponded to 2.1 µM MMC and 28.5 µM quercetin, whereas in WERI-Rb1 cells, they corresponded to 1.8 µM MMC and 35.2 µM quercetin. For combination treatments, drugs were applied at a fixed MMC:quercetin ratio of approximately 1:15, consistent with IC_50_-based ratio determination.

### 4.4. Quantitative Assessment of Drug–Drug Interactions by CI and Isobologram Analysis

Quantitative characterization of the interaction between MMC and quercetin was carried out using the CI approach described by Chou and Talalay, which is based on the median-effect principle and enables objective differentiation among synergistic, additive, and antagonistic interactions [53]. This analytical framework permits evaluation of combination effects across a spectrum of growth-inhibition levels.

For combination analysis, serial dilutions of MMC and quercetin were generated at fixed concentration ratios determined from IC_50_ values obtained in single-agent dose–response experiments. Y79 and WERI-Rb1 cells were treated with these fixed-ratio combinations for 48 h under standard culture conditions, after which viability data obtained from the CCK-8 assay were used as input for interaction assessment.

CI values were derived using CompuSyn software (version 1.0; ComboSyn Inc., Paramus, NJ, USA). In accordance with the Chou–Talalay model, CI values below 1 were interpreted as synergistic interactions, values equal to 1 as additive effects, and values greater than 1 as antagonistic interactions. CI determinations were performed at multiple fractional effect levels, including IC_50_, IC_75_, and IC_90_, to enable comprehensive evaluation of interaction behavior across increasing degrees of growth inhibition.

In parallel with numerical CI analysis, isobologram plots were generated to provide a graphical representation of drug interactions. For each cell line, the doses of MMC and quercetin producing a defined effect level (IC_50_) when applied individually were plotted on the x- and y-axes, respectively, and connected to define the line of additivity. Combination dose pairs achieving the same effect level were subsequently plotted on the same coordinate system. Combination points positioned below the line of additivity were interpreted as synergistic, those located on the line as additive, and those above the line as antagonistic interactions.

### 4.5. PI–FACS Cell Cycle Analysis

Cell cycle distribution was analyzed by flow cytometry using PI staining. Y79 and WERI-Rb1 retinoblastoma cells were treated with MMC and quercetin at concentrations corresponding to their respective IC_50_ values (MMC 2.1 µM and quercetin 28.5 µM for Y79 cells; MMC 1.8 µM and quercetin 35.2 µM for WERI-Rb1 cells) or with their fixed-ratio combination for 48 h under standard culture conditions.

Following treatment, cells were harvested, washed twice with cold phosphate-buffered saline (PBS), and fixed by dropwise addition of 70% ice-cold ethanol while gently vortexing. Fixed cells were stored at −20 °C for at least 12 h to ensure complete permeabilization.

Prior to analysis, cells were washed to remove residual ethanol and incubated with RNase A (100 µg/mL) and PI (50 µg/mL) for 30 min at room temperature in the dark. Flow cytometric acquisition was performed using a 488 nm excitation laser, and a minimum of 20,000 events were collected per sample.

Doublets and cellular debris were excluded by appropriate gating strategies. Cell cycle distribution was determined based on DNA content, and the proportions of cells in the G0/G1, S, and G2/M phases were quantified using FlowJo software (version 10; BD Biosciences, Ashland, OR, USA). Representative DNA content histograms are presented.

### 4.6. Flow Cytometric Detection and Quantification of Apoptotic Cell Death Using Annexin V-FITC/PI Staining

Apoptotic responses induced by MMC and quercetin, administered either individually or in combination, were assessed using Annexin V-FITC/PI double staining followed by flow cytometric evaluation. Y79 and WERI-Rb1 cells were distributed into 6-well culture plates at densities selected to ensure logarithmic growth at the time of treatment. Cells were exposed to MMC, quercetin, or their combination at concentrations corresponding to their respective IC_50_ values (MMC 2.1 µM and quercetin 28.5 µM for Y79 cells; MMC 1.8 µM and quercetin 35.2 µM for WERI-Rb1 cells) and maintained under standard culture conditions for 48 h.

Following treatment, cells were collected by gentle centrifugation and washed twice with cold phosphate-buffered saline to remove residual medium. Cell pellets were subsequently resuspended in Annexin V binding buffer, after which Annexin V-FITC (5 µL) and PI (5 µL) were added to each sample. Samples were gently mixed and incubated for 15 min at room temperature under light-protected conditions to permit adequate staining.

Immediately after staining, samples were subjected to flow cytometric analysis. For each condition, acquisition of at least 20,000 events was performed to ensure statistical robustness. Data collection and analysis were carried out using standard flow cytometry analysis software. Based on fluorescence distribution, cell populations were categorized as viable (Annexin V^−^/PI^−^), early apoptotic (Annexin V^+^/PI^−^), late apoptotic or secondary necrotic (Annexin V^+^/PI^+^), or necrotic (Annexin V^−^/PI^+^).

The overall apoptotic fraction was defined as the combined percentage of early and late apoptotic populations. All experimental conditions were assessed in triplicate, and the entire procedure was independently repeated a minimum of three times to confirm reproducibility.

### 4.7. Quantification of VEGF and IL-6 Secretion by Enzyme-Linked Immunosorbent Assay

The impact of MMC and quercetin treatment on cytokine release was examined by quantifying VEGF and interleukin-6 (IL-6) levels in culture supernatants derived from Y79 cells. Cells were exposed to the indicated single-agent or combination treatments for 48 h under standard culture conditions. Upon completion of the exposure period, culture supernatants were collected with care to prevent cellular carryover.

To eliminate residual cells and debris, supernatants were clarified by low-speed centrifugation (approximately 300× *g* for 5 min at 4 °C). The resulting cell-free fractions were transferred to fresh tubes and processed immediately or stored at −80 °C until analysis. Repeated freeze–thaw cycles were avoided to maintain cytokine stability.

Quantification of VEGF and IL-6 concentrations was performed using commercially available sandwich ELISA kits (R&D Systems, Minneapolis, MN, USA). Standard curves and experimental samples were applied to antibody-coated microplates and incubated for defined periods to allow antigen capture. Following a series of washing steps to remove unbound components, enzyme-linked detection antibodies were introduced, and colorimetric signal development was achieved using the provided substrate solution. The enzymatic reaction was terminated with the stop solution prior to measurement.

Optical density values were recorded using a microplate reader at the wavelength specified for each assay. Cytokine concentrations were calculated by interpolation from standard curves generated in parallel. To account for potential differences in cell number or treatment-related effects on viability, VEGF and IL-6 levels were normalized to total cellular protein content or viable cell counts, as appropriate. All measurements were conducted in triplicate, and experiments were independently repeated at least three times.

### 4.8. Quantitative Analysis of Apoptosis- and Stress-Related Gene Expression by Quantitative Real-Time Polymerase Chain Reaction

Transcriptional alterations associated with apoptotic induction and cellular stress responses were assessed by quantitative real-time polymerase chain reaction analysis. Y79 and WERI-Rb1 cells were exposed to MMC, quercetin, or their combination for 48 h under standard culture conditions, after which total RNA was isolated for subsequent gene expression profiling.

#### 4.8.1. RNA Isolation and Quality Assessment

Total RNA was extracted from treated cells using TRIzol^®^ LS Reagent (Thermo Fisher Scientific, Waltham, MA, USA; Catalog No. 10296028). Following phase separation and RNA recovery, samples were resuspended in RNase-free water and maintained at −80 °C until downstream processing. RNA quantity and purity were evaluated by spectrophotometric analysis, with absorbance measurements obtained at 260 nm and 280 nm using a NanoDrop OneC spectrophotometer (Thermo Fisher Scientific). Only RNA preparations exhibiting A260/A280 ratios within the range of 1.8–2.0 were selected for subsequent gene expression analyses.

#### 4.8.2. cDNA Synthesis

For complementary DNA generation, 1 µg of total RNA per sample was subjected to reverse transcription using the High-Capacity cDNA Reverse Transcription Kit (Thermo Fisher Scientific; Catalog No. 4368814). Reverse transcription reactions were carried out under controlled thermal cycling conditions to ensure consistent and comparable cDNA synthesis across all experimental samples.

#### 4.8.3. Primer Design and qRT-PCR Conditions

Gene-specific primers targeting apoptosis-, cell cycle-, and stress-associated transcripts (Table 1) were generated using the Primer-BLAST platform (https://www.ncbi.nlm.nih.gov/tools/primer-blast/, accessed on 5 February 2026), with reference mRNA sequences retrieved from the NCBI GenBank database. In silico evaluation was performed to confirm primer specificity and to exclude the presence of secondary structures or primer–dimer formation. Quantitative PCR assays were conducted using PowerUp™ SYBR™ Green Master Mix (Thermo Fisher Scientific; Catalog No. A25742) in a total reaction volume of 10 µL, assembled in 96-well optical reaction plates.

Amplification and real-time fluorescence detection were carried out using a real-time PCR system under optimized cycling parameters. For each experimental condition, reactions were performed in technical triplicate to ensure measurement reliability.

#### 4.8.4. Data Analysis

Relative transcript abundance was determined using the comparative cycle threshold approach (2^−ΔΔCt^). Gene expression values were normalized to an internal reference transcript and subsequently scaled relative to untreated control samples, which served as the baseline reference. Final results are reported as fold changes compared with control conditions. All experimental analyses were performed in at least three independent replicates to ensure reproducibility.

### 4.9. Protein–Protein Interaction Network Construction and Pathway Enrichment Analysis

PPI network analysis was employed to examine potential points of convergence between molecular pathways associated with MMC and quercetin, using the STRING database (Search Tool for the Retrieval of Interacting Genes/Proteins; version 12.0). Proteins linked to MMC-related DNA damage responses and quercetin-associated signaling modulation were initially identified through literature-based screening. Network assembly and visualization subsequently focused on regulatory proteins involved in DNA damage response, apoptotic signaling, and cell cycle control that overlapped with experimentally interrogated targets.

The curated protein set was uploaded to the STRING platform, and interaction networks were generated using high-confidence interaction criteria (confidence score > 0.7) to capture experimentally supported and predicted functional associations. The resulting network architecture was examined to delineate shared signaling pathways and biological processes potentially representing points of mechanistic convergence between MMC and quercetin, with particular emphasis on stress-response and apoptosis-related signaling cascades.

To further define the functional context of the interaction network, pathway enrichment analyses were conducted using the Kyoto Encyclopedia of Genes and Genomes (KEGG) and Gene Ontology (GO) databases. Enrichment profiling was applied to identify biological processes, molecular functions, and signaling pathways that were statistically overrepresented among the proteins included in the network. These enrichment outcomes were interpreted alongside experimentally observed cellular responses to aid mechanistic interpretation, rather than serving as independent predictive evidence.

### 4.10. Three-Dimensional Retinoblastoma Tumor Spheroid Experiments

#### 4.10.1. Establishment of 3D Retinoblastoma Tumor Spheroids

A three-dimensional (3D) retinoblastoma spheroid system was established to model treatment responses under conditions that more closely approximate tumor architecture and diffusion constraints encountered in solid tissues. Y79 (ATCC^®^ HTB-18™) and WERI-Rb1 (ATCC^®^ HTB-169™) retinoblastoma cells were cultured in RPMI-1640 medium supplemented with 10% FBS and 1% penicillin–streptomycin. This serum-containing medium supports stable cell aggregation and spheroid formation in retinoblastoma cell lines and has been widely used in previously reported retinoblastoma spheroid culture models.

Cells harvested during the logarithmic growth phase were dispensed into ultra-low attachment round-bottom 96-well plates (Corning Inc., Corning, NY, USA) at a density of 3 × 10^3^ cells per well in a final volume of 200 µL. Plates were maintained at 37 °C in a humidified incubator with 5% CO_2_ to permit spontaneous cell aggregation and spheroid assembly. Compact and uniformly shaped spheroids developed within 48–72 h and were subsequently selected for downstream experimental applications.

#### 4.10.2. Treatment of 3D Tumor Spheroids

Following spheroid formation, treatment conditions were initiated by exchanging the culture medium with fresh medium supplemented with MMC (Sigma-Aldrich, St. Louis, MO, USA), quercetin (Sigma-Aldrich, USA), or their combination. Drug concentrations were selected based on IC_50_ values obtained from two-dimensional monolayer dose–response experiments and subsequently adjusted to compensate for limited compound diffusion within the three-dimensional spheroid structure. Accordingly, MMC and quercetin were applied at concentrations corresponding to their respective IC_50_ values derived from monolayer assays. Vehicle-treated spheroids served as control conditions. To account for the slower growth kinetics and drug penetration characteristics of three-dimensional cultures, all treatments in the spheroid experiments were maintained for 72 h under standard culture conditions.

#### 4.10.3. Bright-Field Imaging and Spheroid Size Measurement

Morphological alterations in three-dimensional tumor spheroids were documented by inverted light microscopy using a digital imaging-equipped system (Olympus, Tokyo, Japan). Bright-field images were captured under standardized optical and acquisition parameters across all experimental conditions to ensure consistency and comparability.

Quantitative assessment of spheroid size was performed using ImageJ software (version 1.53; National Institutes of Health, Bethesda, MD, USA). Spheroid diameter was calculated as the average of two orthogonal measurements obtained at the widest regions of each spheroid. Analyses were conducted on spheroids generated from a minimum of three independent biological experiments.

#### 4.10.4. Evaluation of Cell Viability in 3D Spheroids

Cellular viability within three-dimensional tumor spheroids was evaluated using an ATP-based luminescence assay optimized for 3D culture systems (CellTiter-Glo^®^ 3D Cell Viability Assay; Promega, Madison, WI, USA). To facilitate complete spheroid disruption and ATP release, an equivalent volume of assay reagent was introduced directly into each well. Following reagent addition, plates were processed to allow stabilization of the luminescent signal.

Luminescence output, reflecting intracellular ATP content, was recorded using a microplate reader (BioTek Instruments, Winooski, VT, USA). Viability values were calculated and expressed as percentages relative to vehicle-treated control spheroids.

#### 4.10.5. Live/Dead Fluorescence Visualization of 3D Tumor Spheroids

Treatment-associated cytotoxic effects within three-dimensional spheroids were examined using fluorescence-based viability staining with Calcein-AM and Ethidium homodimer-1 (Thermo Fisher Scientific, Waltham, MA, USA). After completion of the staining procedure, spheroids were visualized by inverted fluorescence microscopy (Olympus, Tokyo, Japan) using appropriate excitation and emission filter sets.

Image acquisition was performed at a uniform focal depth and with identical exposure parameters across all experimental conditions. No post-acquisition image processing or modification was applied, thereby enabling objective visual comparison of viable and non-viable cell populations among treatment groups.

### 4.11. Assessment of ROS-Mediated Cytotoxicity by NAC Pretreatment

The involvement of ROS in treatment-associated cytotoxicity was examined through antioxidant rescue experiments employing N-acetyl-L-cysteine (NAC). Three-dimensional retinoblastoma spheroids were generated as described above and maintained in culture until compact and uniformly shaped structures were established.

Prior to drug exposure, mature spheroids were incubated with NAC at a concentration of 5 mM for 1 h at 37 °C in a humidified atmosphere containing 5% CO_2_. The selected NAC concentration and incubation period were chosen to provide effective antioxidant activity without inducing cytotoxic effects. Following this pretreatment, spheroids were subsequently exposed to MMC, quercetin, or their combination, using the same drug concentrations applied in the corresponding 3D spheroid treatment assays.

Control conditions included untreated spheroids as well as spheroids receiving NAC alone. After completion of the treatment protocol, spheroids were maintained for the indicated time intervals prior to downstream analytical procedures.

#### 4.11.1. Determination of Intracellular ROS Levels Following NAC Pretreatment

Intracellular ROS levels were determined using the fluorescent indicator 2′,7′-dichlorodihydrofluorescein diacetate (DCFH-DA). Upon completion of treatment, three-dimensional spheroids were enzymatically dissociated into single-cell suspensions using Accutase^®^ (Sigma-Aldrich, St. Louis, MO, USA) to enable cell-level fluorescence analysis.

Isolated cells were rinsed with phosphate-buffered saline and subsequently incubated with DCFH-DA at a final concentration of 10 µM for 30 min at 37 °C under light-protected conditions. After incubation, the unincorporated probe was removed by washing with phosphate-buffered saline, and fluorescence signals were immediately acquired by flow cytometric analysis.

ROS-associated fluorescence was quantified as mean fluorescence intensity (MFI). Values were normalized to untreated control samples to allow comparative assessment of intracellular ROS production across experimental conditions.

#### 4.11.2. Evaluation of Cell Viability and Apoptosis Following NAC Pretreatment

The effect of NAC pretreatment on treatment-associated cytotoxicity was evaluated using an ATP-dependent luminescence assay optimized for three-dimensional spheroid cultures (CellTiter-Glo^®^ 3D Cell Viability Assay; Promega, Madison, WI, USA). Following completion of drug exposure, luminescent output reflecting intracellular ATP content was recorded using a microplate reader.

Cell viability values were calculated as percentages relative to untreated control spheroids. The degree to which NAC pretreatment modulated MMC- and quercetin-induced cytotoxicity was subsequently determined. Predictable attenuation of cytotoxic effects following antioxidant exposure was interpreted as evidence supporting a contributory role of ROS in mediating the observed treatment response.

### 4.12. Statistical Analysis

Employed experimental designs incorporated a minimum of three independent biological replicates. Quantitative results are reported as mean values with corresponding standard deviations. Statistical processing and data visualization were performed using GraphPad Prism software (version 9.0; GraphPad Software, San Diego, CA, USA).

Comparisons among multiple experimental conditions were carried out using one-way analysis of variance. Where overall significance was detected, Tukey’s post hoc procedure was applied to enable pairwise group comparisons. Statistical significance was defined using a threshold value of *p* < 0.05. CI determinations for drug interaction analyses were conducted using CompuSyn software, as outlined in the preceding sections.

## 5. Conclusions

The present findings demonstrate that combining quercetin with low-dose MMC elicits a pronounced and reproducible antitumor response in retinoblastoma models. Synergistic cytotoxicity and enhanced apoptotic cell death were consistently observed across two genetically and phenotypically distinct retinoblastoma cell lines, underscoring the robustness of the combination effect. At the mechanistic level, the MMC–quercetin combination is associated with transcriptional changes consistent with p53-related signaling pathways, as evidenced by upregulation of CDKN1A (p21), an increased BAX/BCL-2 ratio, increased CASP3 expression consistent with apoptotic signaling, PI–FACS-defined G2/M cell cycle arrest, and partial involvement of oxidative stress-associated mechanisms, as evidenced by NAC-mediated attenuation of cytotoxicity.

Importantly, the antitumor activity of the MMC–quercetin combination was preserved in a three-dimensional tumor spheroid system, where treatment resulted in marked spheroid shrinkage, disruption of structural integrity, reduced cellular viability, and increased cell death. These findings support the relevance of the observed effects under conditions that more closely approximate in vivo tumor architecture. Antioxidant rescue experiments further revealed that ROS contribute to, but do not fully account for, the observed cytotoxic synergy, as N-acetyl-L-cysteine pretreatment only partially attenuated treatment-induced cell death in three-dimensional spheroids.

Beyond direct cytotoxic effects, the combination was also associated with suppression of tumor-supportive microenvironmental signaling, as reflected by reduced secretion of VEGF and IL-6, suggesting potential anti-angiogenic and immunomodulatory effects. Collectively, these results support the concept of quercetin as a rational chemosensitizing adjuvant that may enhance MMC efficacy. Nevertheless, translation of these findings into clinical application will require rigorous in vivo validation, comprehensive ocular safety evaluation, and optimization of localized ocular drug delivery strategies. However, these findings are based on transcriptional and phenotypic analyses and should be interpreted with caution, requiring further validation through protein-level and in vivo studies.

## Figures and Tables

**Table 1:** Human Gene Primer Sequences Used in qRT-PCR Analysis.

Genes	Primer Sequence (5′ → 3′)
BAX	F: CCCGAGAGGTCTTTTTCC GAG R: CCAGCCCCATGATGGTTCTG
BCL2	F: CATGTGTGTGGAGAGCGTCAA R: CAGATAGGCACCCAGGGTGAT
CASP3	F: CATGGAGCCAAGCCTAAATCTC R: TGTACCAGACCGAGATGTCA
TP53	F: GAGGTTGGCTCTGACTGTACC R: TCCGTGGCAGTCATAGCACT
CDKN1A	F: TGTCCGTCAGAACCCATGC R: AAAGTCGAAGTTCCATCGCTC
HMOX1	F: AAGACTGCGTTCCTGCTCAAC R: GCTCAATCTCCTCCTCCAG
GAPDH	F: GGAGCGAGATCCCTCCAAAAT R: GGCTGTTGTCATACTTCTCATGG

## Data Availability

The original contributions presented in this study are included in the article/Supplementary Material. Further inquiries can be directed to the corresponding author.

## Supplementary Materials

The following supporting information can be downloaded at https://www.mdpi.com/article/10.3390/ph19040545/s1. Figure S1: Dose-dependent effects of MMC on cell viability in Y79 and WERI-Rb1 retinoblastoma cells.

## Abbreviations

MMC	Mitomycin: C
Y79	Y79: retinoblastoma cell line
WERI-Rb1	WERI-Rb1: retinoblastoma cell line
CI	Combination: Index
PI	Propidium iodide
BAX	BCL-2-associated X protein
BCL-2	B-cell lymphoma 2
TP53	Tumor protein p53
CASP3	Caspase 3
CDKN1A	Cyclin-dependent kinase inhibitor 1A
HMOX1	Heme oxygenase 1
PCR	Polymerase chain reaction
VEGF	Vascular endothelial growth factor
IL-6	Interleukin 6
ROS	Reactive oxygen species
NAC	N-acetylcysteine
DNA	Deoxyribonucleic acid
PI3K	Phosphoinositide 3 kinase
AKT	Protein kinase B
MAPK	Mitogen-activated protein kinase
NF-κB	Nuclear factor kappa B
DMSO	Dimethyl sulfoxide
RPMI-1640	Roswell Park Memorial Institute 1640
FBS	Fetal bovine serum
EDTA	Ethylenediaminetetraacetic acid
CCK-8	Cell Counting Kit-8
PBS	Phosphate-buffered saline
ELISA	Enzyme-linked immunosorbent assay
RNA	Ribonucleic acid
NCBI	National Center for Biotechnology Information
PPI	Protein–protein interaction
STRING	Search Tool for the Retrieval of Interacting Genes
KEGG	Kyoto Encyclopedia of Genes and Genomes
GO	Gene Ontology
ANOVA	Analysis of variance
ATP	Adenosine triphosphate
DCFH-DA	Dichlorodihydrofluorescein diacetate
MFI	Mean fluorescence intensity
GAPDH	Glyceraldehyde 3 phosphate dehydrogenase
FDR	False discovery rate
ERK	Extracellular signal-regulated kinase
MDM2	Mouse double minute 2 homolog
G2/M	G2/M phase
CDK1	Cyclin-dependent kinase 1
HIF-1α	Hypoxia inducible factor 1 alpha
MDMX	Mouse double minute X

## References

[1] Dimaras H., Kimani K., Dimba E.A., Gronsdahl P., White A., Chan H.S., Gallie B.L. (2012). Retinoblastoma. Lancet.

[2] Roh J.L., Koo B.S., Yoon Y.H., Rha K.S., Park C.I. (2005). Effect of Topical Mitomycin C on the Healing of Surgical and Laser Wounds: A Hint on Clinical Application. Otolaryngol. Head Neck Surg..

[3] Rishi P., Agarwal A., Chatterjee P., Sharma T., Sharma M., Saravanan M., Ravikumar R. (2020). Intra-Arterial Chemotherapy for Retinoblastoma: Four-Year Results from a Tertiary Center in India. Ocul. Oncol. Pathol..

[4] Golan T., Grenader T., Ohana P., Amitay Y., Shmeeda H., La-Beck N.M., Tahover E., Berger R., Gabizon A.A. (2015). Pegylated Liposomal Mitomycin C Prodrug Enhances Tolerance of Mitomycin C: A Phase 1 Study in Advanced Solid Tumor Patients. Cancer Med..

[5] Tang S.-M., Deng X.-T., Zhou J., Li Q.-P., Ge X.-X., Miao L. (2020). Pharmacological Basis and New Insights of Quercetin Action in Respect to Its Anti-Cancer Effects. Biomed. Pharmacother..

[6] Reyes-Farias M., Carrasco-Pozo C. (2019). The Anti-Cancer Effect of Quercetin: Molecular Implications in Cancer Metabolism. Int. J. Mol. Sci..

[7] Mousavi-Kiasary S.M.S., Senabreh A., Zandi A., Pena R., Cruz F., Adibi A., Hooshmand N. (2025). Synergistic Cancer Therapies Enhanced by Nanoparticles: Advancing Nanomedicine Through Multimodal Strategies. Pharmaceutics.

[8] Panche A.N., Diwan A.D., Chandra S.R. (2016). Flavonoids: An overview. J. Nutr. Sci..

[9] Amawi H., Ashby C.R., Tiwari A.K. (2017). Cancer chemoprevention through dietary flavonoids: What’s limiting?. Chin. J. Cancer.

[10] Huang W.Y., Cai Y.Z., Zhang Y. (2010). Natural phenolic compounds from medicinal herbs and dietary plants: Potential use for cancer prevention. Nutr. Cancer.

[11] Spagnuolo C., Russo G.L., Orhan I.E., Habtemariam S., Daglia M., Sureda A., Nabavi S.F., Devi K.P., Loizzo M.R., Tundis R. (2015). Genistein and cancer: Current status, challenges, and future directions. Adv. Nutr..

[12] Toprak V., Özdemir İ., Öztürk Ş., Yanar O., Kizildemir Y.Z., Tuncer M.C. (2025). Thymoquinone Enhances Doxorubicin Efficacy via RAS/RAF Pathway Modulation in Ovarian Adenocarcinoma. Pharmaceutics.

[13] Karaosmanoğlu Ö., Kamalak Z., Özdemir İ., Öztürk Ş., Tuncer M.C. (2024). Apoptotic Effect of Thymoquinone on OVCAR3 Cells via the P53 and CASP3 Activation. Acta Cir. Bras..

[14] Kashyap D., Garg V.K., Tuli H.S., Yerer M.B., Sak K., Sharma A.K., Kumar M., Aggarwal V., Sandhu S.S. (2019). Fisetin and Quercetin: Promising Flavonoids with Chemopreventive Potential. Biomolecules.

[15] Afşin Y., Alkan Akalın S., Özdemir İ., Tuncer M.C., Öztürk Ş. (2026). Molecular Insights into the Synergistic Anticancer and Oxidative Stress–Modulating Activity of Quercetin and Gemcitabine. Antioxidants.

[16] Faria A., Calhau C., de Freitas V., Mateus N. (2006). Procyanidins as Antioxidants and Tumor Cell Growth Modulators. J. Agric. Food Chem..

[17] Borska S., Chmielewska M., Wysocka T., Drag-Zalesinska M., Zabel M., Dziegiel P. (2012). In Vitro Effect of Quercetin on Human Gastric Carcinoma: Targeting Cancer Cells Death and MDR. Food Chem. Toxicol..

[18] Rana J.N., Mumtaz S. (2025). Prunin: An Emerging Anticancer Flavonoid. Int. J. Mol. Sci..

[19] Rana J.N., Gul K., Mumtaz S. (2025). Isorhamnetin: Reviewing Recent Developments in Anticancer Mechanisms and Nanoformulation-Driven Delivery. Int. J. Mol. Sci..

[20] Asgharian P., Tazekand A.P., Hosseini K., Forouhandeh H., Ghasemnejad T., Ranjbar M., Hasan M., Kumar M., Beirami S.M., Tarhriz V. (2022). Potential mechanisms of quercetin in cancer prevention: Focus on cellular and molecular targets. Cancer Cell Int..

[21] Li Y., Yao J., Han C., Yang J., Chaudhry M.T., Wang S., Liu H., Yin Y. (2016). Quercetin, Inflammation and Immunity. Nutrients.

[22] Khan F., Niaz K., Maqbool F., Ismail Hassan F., Abdollahi M., Nagulapalli Venkata K.C., Nabavi S.M., Bishayee A. (2016). Molecular Targets Underlying the Anticancer Effects of Quercetin: An Update. Nutrients.

[23] Yan S., Yan J., Liu D., Li X., Kang Q., You W., Zhang J., Wang L., Tian Z., Lu W. (2021). A Nano-Predator of Pathological MDMX Construct by Clearable Supramolecular Gold(I)-Thiol-Peptide Complexes Achieves Safe and Potent Anti-Tumor Activity. Theranostics.

[24] Patel A., Anderson J., Kraft D., Finnon R., Finnon P., Scudamore C.L., Manning G., Bulman R., Brown N., Bouffler S. (2016). The Influence of the CTIP Polymorphism, Q418P, on Homologous Recombination and Predisposition to Radiation-Induced Tumorigenesis (Mainly rAML) in Mice. Radiat. Res..

[25] Cheng W., Zhou Y., Chu X., Huang S., Zheng X., Zheng H. (2023). Effect of Intravesical Mitomycin Compared with Gemcitabine on the Treatment Non-Muscle Invasive Bladder Cancer: A Meta-Analysis. Actas Urol. Esp..

[26] Verweij J., Pinedo H.M. (1990). Mitomycin C: Mechanism of Action, Usefulness and Limitations. Anti-Cancer Drugs.

[27] Hasan A.A., Tatarskiy V., Kalinina E. (2022). Synthetic Pathways and the Therapeutic Potential of Quercetin and Curcumin. Int. J. Mol. Sci..

[28] Almatroodi S.A., Alsahli M.A., Almatroudi A., Verma A.K., Aloliqi A., Allemailem K.S., Khan A.A., Rahmani A.H. (2021). Potential Therapeutic Targets of Quercetin, a Plant Flavonol, and Its Role in the Therapy of Various Types of Cancer through the Modulation of Various Cell Signaling Pathways. Molecules.

[29] Kashyap D., Sharma A., Sak K., Tuli H.S., Buttar H.S., Bishayee A. (2018). Fisetin: A Bioactive Phytochemical with Potential for Cancer Prevention and Pharmacotherapy. Life Sci..

[30] Wang P., Zhang K., Zhang Q., Mei J., Chen C.J., Feng Z.Z., Yu D.H. (2012). Effects of Quercetin on the Apoptosis of the Human Gastric Carcinoma Cells. Toxicol. In Vitro.

[31] Czabotar P.E., Lessene G., Strasser A., Adams J.M. (2014). Control of Apoptosis by the BCL-2 Protein Family: Implications for Physiology and Therapy. Nat. Rev. Mol. Cell Biol..

[32] Vazquez A., Bond E.E., Levine A.J., Bond G.L. (2008). The Genetics of the p53 Pathway, Apoptosis and Cancer Therapy. Nat. Rev. Drug Discov..

[33] Singh S.K., Banerjee S., Acosta E.P., Lillard J.W., Singh R. (2017). Resveratrol Induces Cell Cycle Arrest and Apoptosis with Docetaxel in Prostate Cancer Cells via a p53/p21WAF1/CIP1 and p27KIP1 Pathway. Oncotarget.

[34] Nassiri N., Sheibani K., Kavousnezhad S., Nassiri S., Azemati A., Nassiri N. (2025). Use of Mitomycin C in Ophthalmic Surgery. J. Curr. Ophthalmol..

[35] Gartel A.L., Tyner A.L. (2002). The Role of the Cyclin-Dependent Kinase Inhibitor p21 in Apoptosis. Mol. Cancer Ther..

[36] Kastan M.B., Bartek J. (2004). Cell-Cycle Checkpoints and Cancer. Nature.

[37] Lindqvist A., Rodríguez-Bravo V., Medema R.H. (2009). The decision to enter mitosis: Feedback and redundancy in the mitotic entry network. J. Cell Biol..

[38] Smits V.A., Medema R.H. (2001). Checking out the G(2)/M transition. Biochim. Biophys. Acta.

[39] Löbrich M., Jeggo P.A. (2007). The impact of a negligent G2/M checkpoint on genomic instability and cancer induction. Nat. Rev. Cancer.

[40] Boots A.W., Haenen G.R.M.M., Bast A. (2008). Health Effects of Quercetin: From Antioxidant to Nutraceutical. Eur. J. Pharmacol..

[41] Weidner N., Semple J.P., Welch W.R., Folkman J. (1991). Tumor Angiogenesis and Metastasis-Correlation in Invasive Breast Carcinoma. N. Engl. J. Med..

[42] Kumari N., Dwarakanath B.S., Das A., Bhatt A.N. (2016). Role of Interleukin-6 in Cancer Progression and Therapeutic Resistance. Tumour Biol..

[43] Park C., Lee W.S., Go S.-I., Nagappan A., Han M.H., Hong S.H., Kim G.S., Kim G.Y., Kwon T.K., Ryu C.H. (2015). Morin, a Flavonoid from Moraceae, Induces Apoptosis by Induction of BAD Protein in Human Leukemic Cells. Int. J. Mol. Sci..

[44] Comalada M., Camuesco D., Sierra S., Ballester I., Xaus J., Gálvez J., Zarzuelo A. (2005). In Vivo Quercitrin Anti-Inflammatory Effect Involves Release of Quercetin, Which Inhibits Inflammation through Down-Regulation of the NF-κB Pathway. Eur. J. Immunol..

[45] Yousef Y.A., Soliman S.E., Astudillo P.P.P., Durairaj P., Dimaras H., Chan H.S.L., Heon E., Gallie B.L. (2016). Intra-Arterial Chemotherapy for Retinoblastoma: A Systematic Review. JAMA Ophthalmol..

[46] McFall R.C., Sery T.W., Makadon M. (1977). Characterization of a New Continuous Cell Line Derived from a Human Retinoblastoma. Cancer Res..

[47] Kapalczyńska M., Kolenda T., Przybyła W., Zajączkowska M., Teresiak A., Filas V., Ibbs M., Bliźniak R., Łuczewski Ł., Lamperska K. (2018). 2D and 3D Cell Cultures a Comparison of Different Types of Cancer Cell Cultures. Arch. Med. Sci..

[48] Diestelhorst M., Krieglstein G.K. (1995). Ocular Concentrations of Mitomycin C after Extraocular Application in Rabbits. J. Ocul. Pharmacol. Ther..

[49] Attar E.S., Chaudhari V.H., Deokar C.G., Dyawanapelly S., Devarajan P.V. (2023). Nano Drug Delivery Strategies for an Oral Bioenhanced Quercetin Formulation. Eur. J. Drug Metab. Pharmacokinet..

[50] Laurie N.A., Donovan S.L., Shih C.-S., Zhang J., Mills N., Fuller C., Teunisse A., Lam S., Ramos Y., Mohan A. (2006). Inactivation of the p53 Pathway in Retinoblastoma. Nature.

[51] Niazvand F., Orazizadeh M., Khorsandi L., Abbaspour M., Mansouri E., Khodadadi A. (2019). Effects of Quercetin-Loaded Nanoparticles on MCF-7 Human Breast Cancer Cells. Medicina.

[52] Guha P., Dey A., Sen R., Chatterjee M., Chattopadhyay S., Bandyopadhyay S.K. (2011). Intracellular GSH Depletion Triggered Mitochondrial Bax Translocation to Accomplish Resveratrol-Induced Apoptosis in the U937 Cell Line. J. Pharmacol. Exp. Ther..

[53] Chou T.-C. (2010). Drug Combination Studies and Their Synergy Quantification Using the Chou-Talalay Method. Cancer Res..

