# Supplementary material for: Quercetin Sensitizes Retinoblastoma Cells to Mitomycin C Through Transcriptional Modulation of p53-Regulated Apoptotic Genes: A Preclinical Study
Source: Pharmaceuticals (Basel). 2026 Mar 28;19(4):545. doi: 10.3390/ph19040545 (PMC13118558; doi:10.3390/ph19040545)
Supplement: Supplementary file 1 [file pharmaceuticals-19-00545-s001.zip › flow cytometry raw data.pdf]

Representative Flow Cytometry Raw Data – Annexin V / PI

Control (WERI-Rb1)

Quadrant	Population	% Cells	Event Count
Q4	Live	94.1	18820
Q3	Early Apoptosis	3.2	640
Q2	Late Apoptosis	1.8	360
Q1	Necrotic	0.9	180

MMC + Quercetin Treatment (WERI-Rb1)

Quadrant	Population	% Cells	Event Count
Q4	Live	54.0	10800
Q3	Early Apoptosis	23.5	4700
Q2	Late Apoptosis	18.8	3760
Q1	Necrotic	3.7	740

Note: For each sample, 20,000 events were acquired during flow cytometry analysis. Cell populations were classified as live, early apoptotic, late apoptotic, or necrotic based on Annexin V-FITC/PI staining.
