# Supplementary material for: Quercetin Sensitizes Retinoblastoma Cells to Mitomycin C Through Transcriptional Modulation of p53-Regulated Apoptotic Genes: A Preclinical Study
Source: Pharmaceuticals (Basel). 2026 Mar 28;19(4):545. doi: 10.3390/ph19040545 (PMC13118558; doi:10.3390/ph19040545)
Supplement: Supplementary file 1 [file pharmaceuticals-19-00545-s001.zip › Raw data for Figure 1.pdf]

Raw Data

They represent replicate measurements (Rep1–Rep3).

MMC – Y79 (48 h)

Concentration (µM)	Rep1	Rep2	Rep3	Mean
0.01	100	99	100	99.7
0.1	97	96	98	97
0.3	91	89	90	90
1	72	69	70	70.3
3	42	39	40	40.3
10	16	14	15	15

MMC – WERI-Rb1 (48 h)

Concentration (µM)	Rep1	Rep2	Rep3	Mean
0.01	100	99	100	99.7
0.1	97	98	96	97
0.3	89	87	88	88
1	66	64	65	65
3	33	31	32	32
10	13	11	12	12

Quercetin – Y79 (48 h)

Concentration (µM)	Rep1	Rep2	Rep3	Mean
10	91	89	90	90
20	76	74	75	75
30	61	59	60	60

50	41	39	40	40
80	26	24	25	25
100	21	19	20	20

Quercetin – WERI-Rb1 (48 h)

Concentration (µM)	Rep1	Rep2	Rep3	Mean
10	93	91	92	92
20	81	79	80	80
30	66	64	65	65
50	46	44	45	45
80	31	29	30	30
100	26	24	25	25
