# Supplementary material for: Quercetin Sensitizes Retinoblastoma Cells to Mitomycin C Through Transcriptional Modulation of p53-Regulated Apoptotic Genes: A Preclinical Study
Source: Pharmaceuticals (Basel). 2026 Mar 28;19(4):545. doi: 10.3390/ph19040545 (PMC13118558; doi:10.3390/ph19040545)
Supplement: Supplementary file 1 [file pharmaceuticals-19-00545-s001.zip › Raw data for Figure 11.pdf]

Below is the raw data corresponding to the quantitative panels (B and C) of the figure: Spheroid Diameter and Cell Viability for 3D retinoblastoma spheroids.
Format is replicate measurements (n = 3) that generate the bar graphs.

B. Spheroid Diameter Raw Data (µm)

Treatment	Rep1	Rep2	Rep3	Mean	SD
Control	565	575	570	570	5
Quercetin	510	525	520	518	7.6
MMC	440	455	450	448	7.6
Quercetin + MMC	410	425	420	418	7.6

C. Cell Viability Raw Data (% of Control)

Treatment	Rep1	Rep2	Rep3	Mean	SD
Control	100	100	100	100	0
Quercetin	88	90	89	89	1
MMC	79	82	80	80.3	1.5
Quercetin + MMC	72	74	73	73	1

D. Live/Dead Staining Quantification (from fluorescence images)

Typical quantification derived from image analysis:

Treatment	Replicate	Live Cells (%)	Dead Cells (%)
Control	Rep1	96.2	3.8
Control	Rep2	95.7	4.3
Control	Rep3	96.5	3.5
Quercetin + MMC	Rep1	68.4	31.6
Quercetin + MMC	Rep2	66.9	33.1
Quercetin + MMC	Rep3	67.5	32.5
