# Supplementary material for: Quercetin Sensitizes Retinoblastoma Cells to Mitomycin C Through Transcriptional Modulation of p53-Regulated Apoptotic Genes: A Preclinical Study
Source: Pharmaceuticals (Basel). 2026 Mar 28;19(4):545. doi: 10.3390/ph19040545 (PMC13118558; doi:10.3390/ph19040545)
Supplement: Supplementary file 1 [file pharmaceuticals-19-00545-s001.zip › Raw data for Figure 12.pdf]

Below is the raw data tables consistent with the two bar graphs in the figure: ROS generation (DCF-MFI) and cell viability (% of control). Format is replicate measurements (n = 3) that produce the shown means and error bars.

A. Raw Data – Intracellular ROS (Relative DCF-MFI)

Treatment	Rep1	Rep2	Rep3	Mean	SD
Control	103	108	106	105.7	2.5
Quercetin	97	101	100	99.3	2.1
MMC	158	163	160	160.3	2.5
Quercetin + MMC	134	139	137	136.7	2.5
NAC + Quercetin + MMC	106	111	109	108.7	2.5

B. Raw Data – Cell Viability (% of Control)

Treatment	Rep1	Rep2	Rep3	Mean	SD
Control	95	98	97	96.7	1.5
Quercetin	78	82	80	80.0	2.0
MMC	60	63	62	61.7	1.5
Quercetin + MMC	53	55	54	54.0	1.0
NAC + Quercetin + MMC	66	69	68	67.7	1.5
