# Supplementary material for: Quercetin Sensitizes Retinoblastoma Cells to Mitomycin C Through Transcriptional Modulation of p53-Regulated Apoptotic Genes: A Preclinical Study
Source: Pharmaceuticals (Basel). 2026 Mar 28;19(4):545. doi: 10.3390/ph19040545 (PMC13118558; doi:10.3390/ph19040545)
Supplement: Supplementary file 1 [file pharmaceuticals-19-00545-s001.zip › Raw data for Figure 2.pdf]

Below are the raw data. These correspond to the CI–Fa plot and the isobologram analysis for the MMC–quercetin combination (48 h).

A. CI–Fa Plot Raw Data (48 h)

Y79 Cell Line

Fraction Affected (Fa)	Combination Index (CI)
0.50	0.72
0.75	0.65
0.90	0.58

WERI-Rb1 Cell Line

Fraction Affected (Fa)	Combination Index (CI)
0.50	0.81
0.75	0.70
0.90	0.63

Additive Reference Line

Fraction Affected (Fa)	CI
0.50	1.00
0.75	1.00
0.90	1.00

Interpretation reference:

- **CI < 1 → synergistic effect**
- **CI = 1 → additive effect**
- **CI > 1 → antagonistic effect**

B. Isobologram Raw Data (IC_{50} Combination, 48 h)

Single-Agent IC_{50} Values

Cell Line	MMC IC_{50} (μM)	Quercetin IC_{50} (μM)
Y79	2.1	28.5
WERI-Rb1	1.8	35.2

Y79 Combination Doses

MMC (µM)	Quercetin (µM)
0.60	9.0
0.75	12.0
1.00	15.0

WERI-Rb1 Combination Doses

MMC (µM)	Quercetin (µM)
0.70	10.5
0.90	13.5
1.10	16.5
