# Supplementary material for: Quercetin Sensitizes Retinoblastoma Cells to Mitomycin C Through Transcriptional Modulation of p53-Regulated Apoptotic Genes: A Preclinical Study
Source: Pharmaceuticals (Basel). 2026 Mar 28;19(4):545. doi: 10.3390/ph19040545 (PMC13118558; doi:10.3390/ph19040545)
Supplement: Supplementary file 1 [file pharmaceuticals-19-00545-s001.zip › Raw data for Figure 3.pdf]

Raw Data Tables – PI-FACS Cell Cycle and Annexin V/PI Apoptosis (48 h)

PI-FACS Cell Cycle Distribution – Y79

Treatment	Replicate	G0/G1 (%)	S (%)	G2/M (%)
Control	Rep1	55.1	26.2	18.7
Control	Rep2	57.9	24.3	17.8
Control	Rep3	55.6	25.8	18.6
MMC	Rep1	39.2	21.1	39.7
MMC	Rep2	37.6	22.4	40.0
MMC	Rep3	39.3	22.2	38.5
Quercetin	Rep1	41.3	25.2	33.5
Quercetin	Rep2	43.0	24.1	32.9
Quercetin	Rep3	42.0	25.0	33.0
MMC + Quercetin	Rep1	23.5	18.2	58.3
MMC + Quercetin	Rep2	26.1	17.0	56.9
MMC + Quercetin	Rep3	25.2	18.2	56.6

Annexin V / PI Apoptosis – Y79

Treatment	Replicate	Live (%)	Early Apoptosis (%)	Late Apoptosis (%)	Necrotic (%)
Control	Rep1	94.1	2.9	2.0	1.0
Control	Rep2	92.8	3.5	2.4	1.3
Control	Rep3	93.6	3.0	2.3	1.1
MMC	Rep1	71.8	15.1	10.6	2.5
MMC	Rep2	73.2	14.3	10.1	2.4

MMC	Rep3	72.1	14.5	11.0	2.4
Quercetin	Rep1	75.6	13.1	8.8	2.5
Quercetin	Rep2	77.4	12.4	7.9	2.3
Quercetin	Rep3	75.9	13.2	8.4	2.5
Combination	Rep1	51.6	24.2	20.1	4.1
Combination	Rep2	54.8	22.5	19.0	3.7
Combination	Rep3	52.0	23.7	19.1	5.2
