# Supplementary material for: Quercetin Sensitizes Retinoblastoma Cells to Mitomycin C Through Transcriptional Modulation of p53-Regulated Apoptotic Genes: A Preclinical Study
Source: Pharmaceuticals (Basel). 2026 Mar 28;19(4):545. doi: 10.3390/ph19040545 (PMC13118558; doi:10.3390/ph19040545)
Supplement: Supplementary file 1 [file pharmaceuticals-19-00545-s001.zip › Raw data for Figure 4.pdf]

Below is the raw data table format corresponding to the Annexin V/PI density plots (WERI-Rb1, 20,000 events).

Annexin V / PI Apoptosis Raw Data – WERI-Rb1 (48 h)

Treatment	Replicate	Live (Q4, %)	Early Apoptosis (Q3, %)	Late Apoptosis (Q2, %)	Necrotic (Q1, %)
Control	Rep1	94.3	2.8	2.1	0.8
Control	Rep2	93.7	3.1	2.2	1.0
Control	Rep3	94.0	2.9	2.0	1.1
MMC	Rep1	73.5	13.9	9.8	2.8
MMC	Rep2	74.1	13.4	9.6	2.9
MMC	Rep3	72.8	14.1	10.0	3.1
Quercetin	Rep1	76.2	12.5	8.7	2.6
Quercetin	Rep2	77.0	11.9	8.4	2.7
Quercetin	Rep3	75.8	12.7	8.9	2.6
Combination	Rep1	53.4	23.1	18.6	4.9
Combination	Rep2	55.1	22.4	17.8	4.7
Combination	Rep3	54.0	23.0	18.1	4.9

Quadrant definitions

- **Q4 (Annexin− / PI−)** → Live cells
- **Q3 (Annexin+ / PI−)** → Early apoptosis
- **Q2 (Annexin+ / PI+)** → Late apoptosis
- **Q1 (Annexin− / PI+)** → Necrotic cells
