# Supplementary figures and images for: Quercetin Sensitizes Retinoblastoma Cells to Mitomycin C Through Transcriptional Modulation of p53-Regulated Apoptotic Genes: A Preclinical Study
Source: Pharmaceuticals (Basel). 2026 Mar 28;19(4):545. doi: 10.3390/ph19040545 (PMC13118558; doi:10.3390/ph19040545)

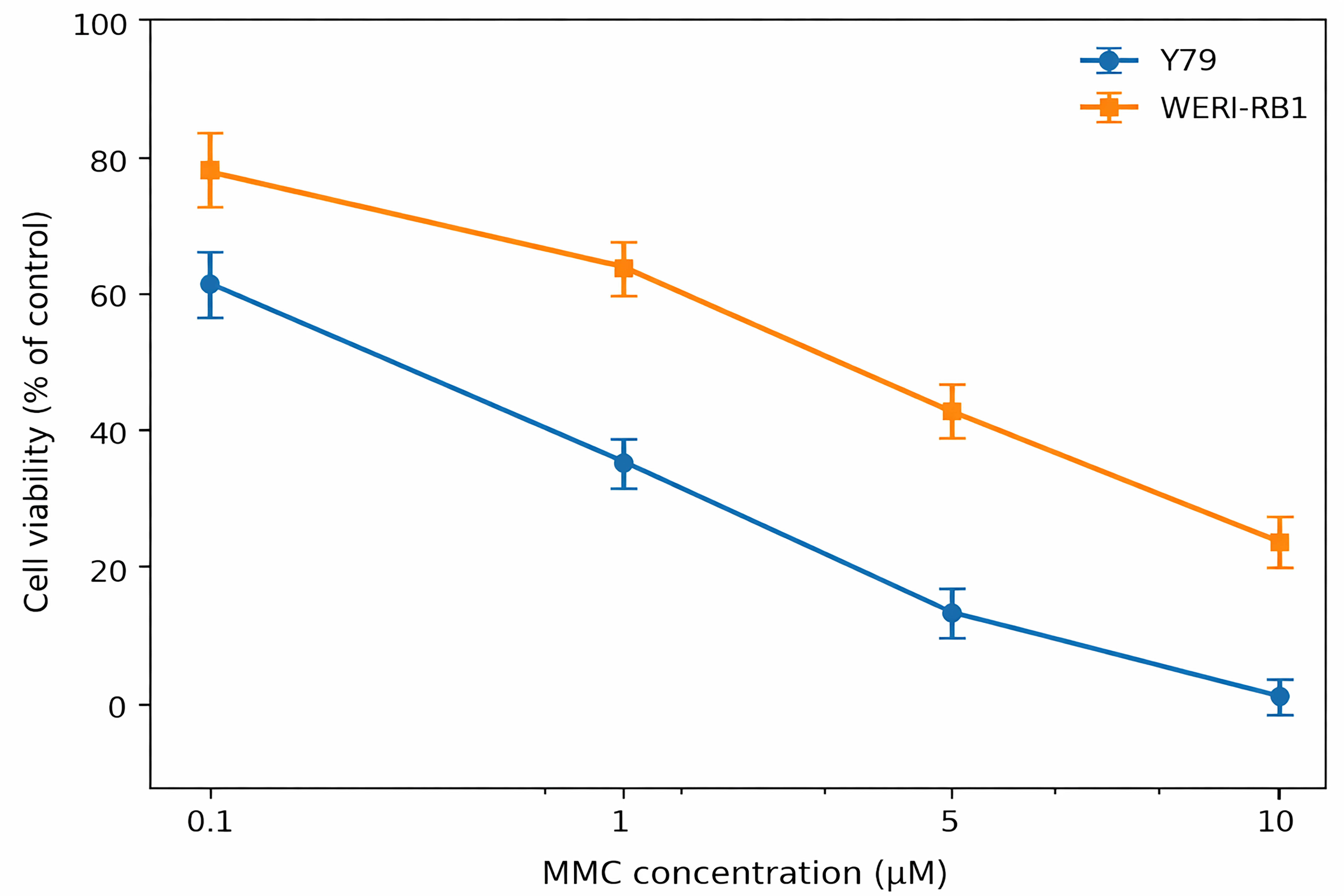

Supplement: Supplementary file 1 [file pharmaceuticals-19-00545-s001.zip › Figure S1.jpg]
